# Research Advances and Disease Modeling in Respiratory Organoids

**DOI:** 10.3390/biomedicines14010221

**Published:** 2026-01-20

**Authors:** Lanhe Chu, Dian Chen, Simin Jiang, Huanyu Long, Xiaojuan Liu, Yahong Chen

**Affiliations:** 1 Department of Pulmonary and Critical Care Medicine, Peking University Third Hospital, 49 Huayuan North Road, Haidian District, Beijing 100191, China; 18377792128@163.com (L.C.); chendian@stu.pku.edu.cn (D.C.); jsm0000@163.com (S.J.); longhuanyu97@163.com (H.L.); 2 Clinical Stem Cell Research Center, Peking University Third Hospital, Beijing 100191, China

**Keywords:** respiratory organoids, disease modeling, applications

## Abstract

Organoid culture represents a sophisticated biological model that surpasses traditional two-dimensional (2D) methods and animal models in physiological relevance and cost-effectiveness. Current organoid systems derive from adult, fetal, and induced pluripotent stem cells, providing innovative platforms for studying organ development, disease pathogenesis, and drug discovery. Recent technological advances now enable respiratory organoids to significantly contribute to respiratory disease research. This review comprehensively synthesizes the development of respiratory organoid models and their applications in studying major respiratory diseases, including pulmonary fibrosis, chronic obstructive pulmonary disease (COPD), and lung cancer. It further evaluates the transformative potential of these models in advancing respiratory disease research. Respiratory organoids uniquely model disease mechanisms and drug responses in human-specific microenvironments, enabling pathogenesis studies of respiratory diseases. They serve as functional platforms for drug screening and personalized therapy development. Future integration of multi-organoid systems with precision medicine promises to redefine respiratory disease research paradigms.

## 1. Introduction

Organoids were originally described as aberrant cell growth or intracellular structures in early three-dimensional (3D) cell culture studies [1]. As research proceeded, the concept was expanded to refer to 3D culture systems derived from stem cells or tissue-specific cells that can self-organize and differentiate into structures mimicking the architecture and function of real organs [2]. These “mini-organs” have become powerful tools for exploring human biology, disease mechanisms, and therapeutic strategies. In healthcare, organoids are widely used in disease modeling, drug discovery and screening, precision medicine, and regenerative medicine. In basic research, they facilitate studies of development, organ function, and pathology. By recapitulating key features of native organs, organoids provide an invaluable platform for advancing biomedical science [3].

2D cell/tissue culture models have long been used to study human diseases and development, yet they are limited by their lack of physiological relevance. In vivo, cells exist within complex microenvironments and are influenced by diverse biochemical and mechanical cues—conditions that 2D cultures fail to reproduce [3,4,5,6]. Consequently, cells in 2D cultures gradually lose their native morphology and heterogeneity, and long-term passaging leads to marked genomic and metabolic divergence from the original cell lines. Animal models such as genetically engineered mouse models (GEMMs) and patient-derived xenografts (PDXs) offer improved physiological fidelity but are costly, time-consuming, and limited by interspecies differences [7,8]. Organoid culture systems overcome many of these drawbacks. Derived from stem or tissue-specific cells, organoids retain the genetic and functional traits of their tissue of origin while maintaining long-term stability in vitro [9,10,11]. By mimicking the in vivo microenvironment, they provide a superior platform for studying disease mechanisms and therapeutic responses.

**Figure 1:**
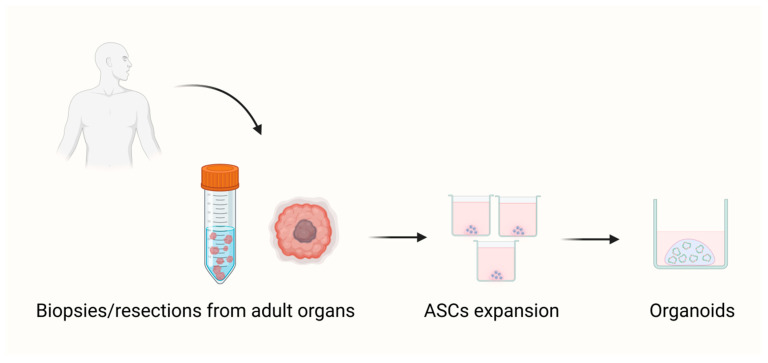
Organoid models derived from adult stem cells. (Created by the authors based on literature data.) ASCs are undifferentiated cells found in adult tissues. ASCs can be isolated from healthy organs or from tumors arising within them. When cultured under appropriate conditions, these ASCs can self-organize to form organoids in vitro.

**Figure 2:**
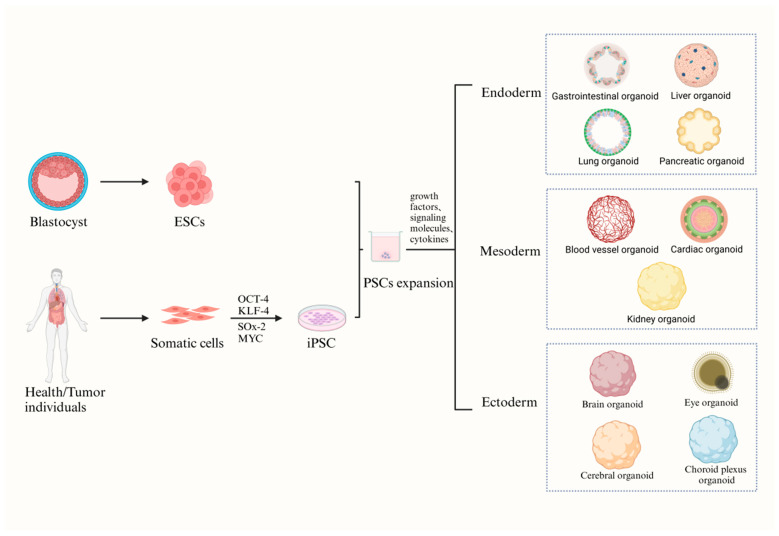
Organoid models derived from fetal and induced pluripotent stem cells. (Adapted from Ref. [3] licensed under CC BY 4.0.) By introducing four transcription factors, somatic cells can be transformed into induced pluripotent stem cells (iPSCs). Both ESCs and iPSCs exhibit pluripotency, enabling differentiation into multiple lineages. Organoid generation from PSCs relies on directed differentiation, in which growth factors, signaling molecules, and cytokines are applied to induce the formation of specific germ layers, including the endoderm, mesoderm, and ectoderm.

Thus far, three different types of organoids have been described based on the source of their starting material: (a) adult stem cells (ASCs), (b) embryonic stem cells (ESCs), and (c) induced pluripotent stem cells (iPSCs) (Figure 1 and Figure 2).

Both ESC/iPSC and ASC-derived organoids can form 3D organ-like structures that partially recapitulate the architecture and function of native organs, though important differences remain (Table 1). Together, these systems provide complementary platforms for scientific research and personalized medicine. Ongoing efforts aim to establish diverse ESC/iPSC- and ASC-based “human disease-in-a-dish” models.

Respiratory organoids, as an emerging 3D model, offer distinct advantages for studying respiratory biology. Unlike traditional 2D lung cell cultures that form basic epithelial layers with progenitor traits, organoids better capture complex cell interactions, extracellular matrix, and physiological signals [25,26,27]. ALI (Air-Liquid Interface) culture system is a valuable in vitro model that effectively mimics the airway epithelial cell microenvironment [28]. However, it has significant limitations, including a limited source of primary cells, a complex culture process, and low expansion capacity. Primary airway basal stem cells (ABSCs) exhibit poor passaging stability, with their differentiation ability declining after 2–3 passages, which restricts long-term experimentation [29]. Additionally, the model’s complexity and reliance on transwell membranes hinder scalability for large applications [30]. Respiratory organoids derived from ASCs offer a short culture duration and long-term maintenance, closely mimicking adult tissues, which is advantageous for regenerative medicine and chronic disease modeling [31]. In contrast, organoids from iPSCs involve more complex, time-consuming, and costly processes, often yielding less mature, fetal-like cells [31]. Due to their diversity, scalability, and reproducibility, respiratory organoids are promising for studying respiratory disease mechanisms, lung regeneration, and personalized therapies [17,19,32,33]. In this review, we summarize recent advances in respiratory organoid technology and its applications in respiratory diseases, highlighting future opportunities in this rapidly evolving field.

## 2. Composition of the Respiratory System and Modeling of Respiratory Organoids

The respiratory system is a complex organ system that can be divided into the upper respiratory tract (URT) and lower respiratory tract (LRT). The URT includes the nostrils, nasal cavity, sinuses, nasopharynx, oropharynx, and the portion of the larynx above the vocal cords, while the LRT comprises the larynx below the vocal cords, trachea, bronchi, bronchioles, and alveoli [34]. Its primary physiological function is the exchange of oxygen and carbon dioxide [35]. The respiratory tract exhibits region-specific cellular compositions and physiological characteristics. Airway epithelium from the nasal cavity to the terminal bronchioles consists mainly of four cell types: ciliated, goblet, basal, and Club (Clara) cells. Together, they maintain mucociliary clearance and protect against inhaled particles and pathogens [36,37]. The epithelial cells of the URT form the first barrier against environmental agents and pathogens. Mucus secreted by glands and goblet cells forms the airway surface liquid (ASL), which supports mucociliary clearance; this process is more efficient in the URT due to its higher density of ciliated cells [37,38]. The LRT epithelium is more complex. In addition to the aforementioned cells, secretory cells regulate airway hydration, maintaining proper mucus flow and clearance [39,40]. The alveoli, as the functional units of gas exchange, are composed of type I (AT1) and type II (AT2) alveolar epithelial cells [41,42,43]. AT1 cells cover about 96% of the alveolar surface and form a very thin blood-gas barrier for efficient exchange [44], while AT2 cells secrete surfactant to reduce surface tension and prevent alveolar collapse [41,43]. AT2 cells also function as facultative progenitor cells, capable of self-renewing and differentiating into AT1 cells during repair [45,46].

During embryonic development, respiratory epithelial cells originate from the endoderm and eventually develop into multiple airway compartments, including the alveoli, bronchioles, and proximal airways [47]. These regions contain specific stem and progenitor cell populations, such as basal cells, Club cells, bronchioalveolar stem cells (BASCs), pulmonary neuroendocrine cells (PNECs), and AT2 cells, that collectively sustain repair and homeostasis [47,48,49]. Basal cells are abundant in the bronchi and gradually decrease distally [50,51]. Club cells are primarily located in distal airways and possess secretory functions [52]. Basal cells exhibit strong proliferative capacity and can differentiate into ciliated, goblet, and Club cells after injury [53,54]. Under steady-state conditions, Club cells show limited self-renewal ability but can differentiate into ciliated and goblet cells; in response to viral infection, they can dedifferentiate into basal cells [55,56,57]. Currently, organoids derived from basal cells, including tracheal, bronchial, and nasal organoids, have been successfully established, containing multiple airway cell types [58,59,60]. Furthermore, BASCs at the mouse bronchial-alveolar junction co-express Club cell markers (Club Cell Secretory Protein, CCSP) and AT2 cell marker (surfactant protein C, SPC). These cells possess self-renewal abilities and can differentiate into Club cells, AT1 cells, and AT2 cells under both homeostatic and injury conditions [61,62]. PNECs emerge during early lung development, initially appearing in proximal conducting airways and later in distal branches, characterized by calcitonin gene-related peptide (CGRP) expression [63]. Lineage tracing studies in mice have revealed that PNECs can self-renew and differentiate into Club and ciliated cells following injury, although their removal does not markedly affect regeneration, suggesting additional repair mechanisms exist [64]. A variant form of Club cells (vClub) in the bronchioles can self-renew and replenish lost Club cells [65]. Finally, AT2 cells in the distal lung, owing to their robust self-renewal and differentiation capacities, serve as ideal sources for generating alveolar-like organoids [66].

**Figure 3:**
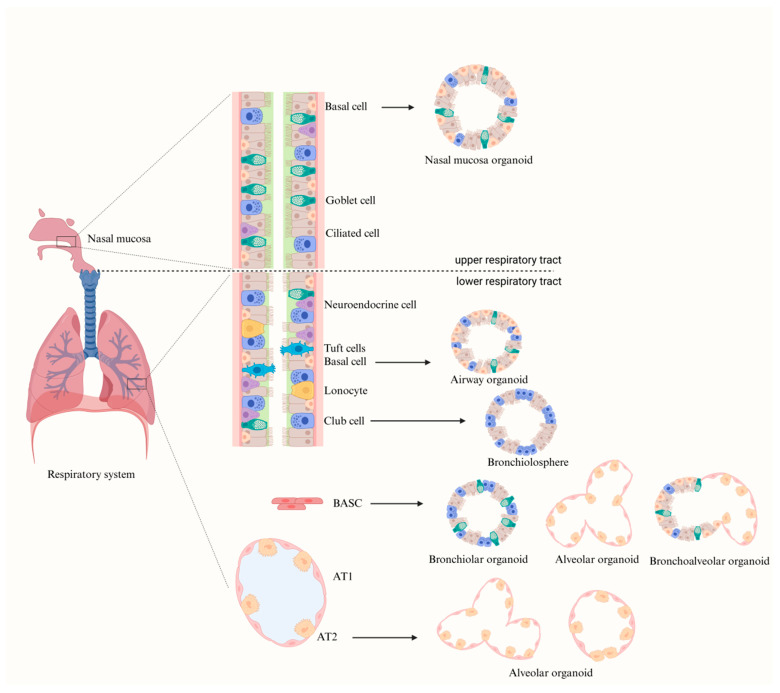
Respiratory organoid models derived from ASCs. (Created by the authors based on literature data.) Illustrated are various airway epithelial cells in the respiratory tract. Basal cells can be used to generate nasal organoids or airway organoids containing basal, ciliated, and secretory cells. Club cells can be used to develop bronchiolospheres containing Club and ciliated cells. Mouse bronchioalveolar stem cells (BASCs) can form alveolar organoids, and they can also generate bronchoalveolar organoids containing tubular-like structures with basal, club, goblet, and ciliated cells and saccular-like structures composed of differentiated AT1 and AT2 cells. Lastly, AT2 cells lead to the formation of alveolar organoids, containing AT1 and AT2 cells.

**Figure 4:**
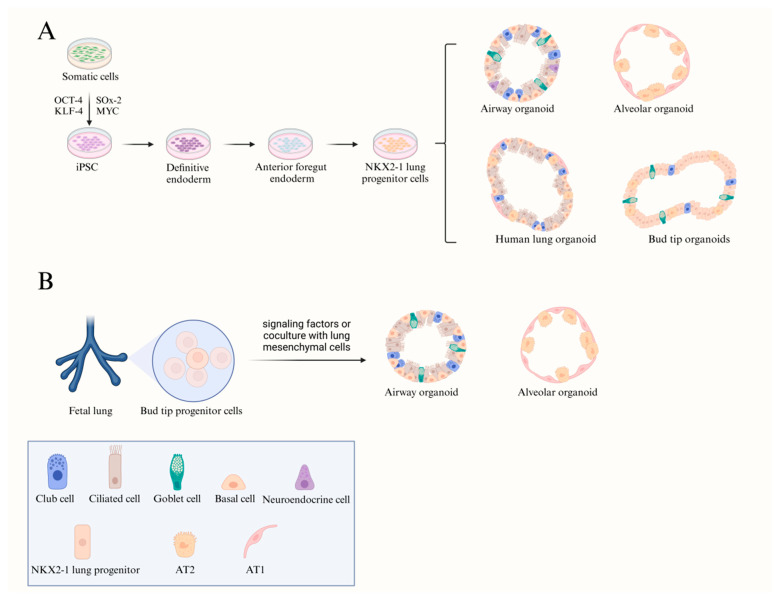
Respiratory organoid models derived from iPSCs and FSCs. (Created by the authors based on literature data.) (**A**) Somatic cells can be reprogrammed into iPSCs through the introduction of four specific transcription factors. Subsequently, these iPSCs can differentiate into airway organoids, alveolar organoids, human lung organoids comprising both alveolar-like cell types and airway-like cell types, and bud tip organoids, by progressing through the developmental stages of definitive endoderm, anterior foregut endoderm, and *NKX2-1^+^* lung progenitor cells. (**B**) Bud tip progenitor cells obtained from fetal lung tissue can be directly differentiated into alveolar and airway organoids under various signaling factors or cocultured with lung mesenchymal cells.

The specific stem and progenitor cell populations in the respiratory epithelium can generate 3D structures that closely recapitulate in vivo cellular organization and interactions through differentiation or co-culture. These structures include nasal, trachea, bronchi, and alveolar organoids. Currently, three types of stem cells are commonly used for constructing respiratory organoids: ASCs (Figure 3), iPSCs, and fetal lung stem cells (FSCs) (Figure 4). Such models are widely used to study respiratory pathophysiology and to explore novel therapeutic strategies.

### 2.1. Nasal Mucosa Organoid

Early investigations predominantly utilized traditional cell lines, such as primary human nasal epithelial (HNE) cells, to examine nasal diseases. However, these models often failed to accurately reflect the physiological characteristics of the human nasal epithelium [67]. With advancements in cell culture methodologies, researchers have successfully reconstructed 3D nasal epithelium in vitro, providing new platforms for studying respiratory viral infections and other airway diseases. Liu et al. developed an in vitro organoid model of human nasal epithelial cells using nasal biopsy samples and characterized its differentiation and imaging features [67]. This model has been employed in cystic fibrosis research and other respiratory disease studies. Following this, Brewington et al. [68] and Gamage et al. [69] also utilized nasal biopsy samples and nasal brushings to create 3D models of nasal epithelial cells. However, these methods are invasive, requiring clinical sampling of nasal or airway tissues, which limits their accessibility for routine research and drug screening. To overcome this, Chiu et al. developed a non-invasive two-phase culture system using nasal epithelial cells from inferior turbinate swabs of healthy donors to model *SARS-CoV-2* infection [70]. This system involves an amplification phase for long-term expansion (over six months) and a differentiation phase to generate mature nasal organoids or 2D monolayers. The differentiated organoids contained ciliated cells, basal cells, goblet cells, and club cells, and expressed high levels of *ACE2*, faithfully mimicking native nasal epithelium. Moreover, adjusting the differentiation medium to mildly acidic conditions (pH 6.6) enhanced epithelial barrier formation, closely simulating the physiological nasal surface environment. Another group established a fully non-invasive human nasal mucosa organoid (HNO) model applicable to both pediatric and adult donors [71]. Stem cells isolated from nasal lavage or turbinate swabs were cultured in 3D and differentiated under air–liquid interface (ALI) conditions, forming polarized, pseudostratified epithelia with ciliated, basal, secretory, and goblet cells. The cultures exhibited motile cilia, maintained long-term proliferative capacity, and could be cryopreserved, making them valuable for respiratory disease modeling. An “amplification-differentiation” culture approach has also been developed using human nasal polyp samples [72]. Following sequential amplification (7 days) and differentiation (14 days), this method generated organoids closely resembling normal nasal mucosa, providing a promising tool for studying nasal disease pathogenesis and drug screening [73]. However, conventional organoid cultures using Matrigel limit cell migration and promote inward growth, resulting in apical surfaces being enclosed within spheroids [30,74]. This orientation may not fully recapitulate the in vivo epithelial structure, as the apical surface of airway epithelium should face the atmosphere and interact with the contents of the lumen. To address this, researchers developed a human nasal mucosa organoid model with an outward-facing apical surface using nasal progenitor cells embedded in a composite hydrogel (CAH gel). This system accurately reproduced epithelial polarity, cellular diversity, and revealed that epithelial-derived matrix metalloproteinases (MMPs) regulate epithelial fate and polarity [75]. Since basal cells are also present in nasal epithelium, organoids or “nasal balls” can be derived from these cells, offering a convenient patient-derived platform for drug screening. Nonetheless, organoid characteristics may vary depending on the anatomical origin of the basal cells within the nasal cavity [76].

### 2.2. Airway Organoid

Nasal organoids, derived from donor nasal epithelial cells, contain major airway epithelial cell types and share similarities with airway organoids. However, nasal mucosa organoids retain the unique characteristics of the upper airway epithelium. In 2016, Satoshi Konishi et al. established the first stepwise 3D differentiation protocol to generate multiciliated airway cells (MCACs) from hiPSCs [77]. Using carboxypeptidase M (CPM) to isolate ventral foregut endodermal cells, they formed 3D progenitor aggregates (‘spheroids’). These airway progenitor spheroids could then be directed to differentiate into a complex 3D epithelium containing MCACs and other airway lineages (excluding alveolar cells). Then the researchers identified optimal culture conditions for proliferating spheroids and inducing *FOXJ1* expression (a representative marker for MCACs), which included FGF10, CHIR99021 (a WNT agonist), KGF, and DAPT (a γ-secretase inhibitor that blocks the Notch pathway). This pioneering work paves the way for future applications aimed at modeling airway diseases or developing airway reconstruction methods, such as artificial tracheas. Subsequent studies revealed that high concentrations of FGF-10 could induce the generation of human lung organoids (HLOs) from hiPSCs. These organoids contained airway-like epithelial components marked by *TP63* and *FOXJ1*, as well as mesenchymal and epithelial cells that simultaneously expressed *SFTPC* and *HOPX*, which are markers of alveolar cells. In contrast, a culture system based on FGF-7, CHIR, and ATRA could induce lung progenitor cells to form bud-tip-like organoids (BTOs) with airway-like regions expressing *SOX2^+^*, *MUC5AC^+^*, and *SCGB1A1^+^*, as well as tip-like structures expressing *SOX2^+^*, *SOX9^+^*, *SFTPC^+^*, and *ID2^+^* [78]. Based on these findings, *SOX2^+^*, *SOX9^+^*, *CPM^+^*^,^ and *NKX2-1^+^* tip progenitor cells were directed to form airway organoids [79]. Nikolić and colleagues et al. later developed fetal lung bud tip (LBT)–derived organoid cultures using a combination of growth factors (EGF, FGF7, FGF10), *BMP* inhibitors (Noggin, SB431542), and *WNT* activators (RSPO1, CHIR) [80]. These maintained *NKX2-1* expression and lung progenitor identity without full differentiation. Subsequent exposure to airway differentiation media or modulation of *SMAD* signaling (via TGF-β, BMP4, A8301, and Noggin) produced proximal airway organoids containing basal, goblet, Club, and ciliated cells [81].

In 2017, Tan et al. [82] first reported the generation of airway organoids by co-culturing human bronchial epithelial cells, lung fibroblasts, and pulmonary microvascular endothelial cells. They found that randomly seeded mixed cell populations could self-organize into branching epithelial-endothelial structures. In 2018, the same group established the first adult stem cell-derived airway organoids from normal lung tissue. These organoids, composed exclusively of epithelial cells, were capable of stable long-term expansion for over a year in an expansion medium containing key growth factors R-spondin, Noggin, FGF7, and FGF10 [30]. Based on a differentiation protocol previously reported by Satoshi Konishi et al. [77], they induced proximal differentiation in these continuously expanding organoids, thereby generating airway organoids that could mimic the airway epithelium. The team continued to explore distal differentiation systems for alveolar cultures, successfully establishing a respiratory organoid culture system with bidirectional differentiation potential for both proximal and distal airway regions [70]. Additionally, Sachs et al. developed a long-term expanding human airway organoid model from human bronchoalveolar lavage fluid and lung tissue specimens from cystic fibrosis and lung cancer patients [83]. By modulating *TGF-β*, *FGF*, and *WNT* signaling pathways, these basal cell-derived airway organoids contained basal cells, ciliated, and secretory/Club cells, and maintained stability for over a year. They preserved tissue pathological features and cancer gene mutations, making them suitable as in vitro models for malignant tumors and infectious lung diseases. Notably, they further optimized the organoid culture system by removing Noggin from the medium and adding DAPT/BMP4 to the standard airway organoid culture media [84]. Researchers also validated the regenerative and multipotent properties of basal cells in bronchial organoids derived from human bronchial tissue. Another study reported that basal cells derived from human bronchial tissue could differentiate into other cell types, including goblet cells and ciliated cells, in bronchial organoids. This differentiation ability was found to be closely associated with the *Notch* signaling pathway [85]. Co-culturing Club cells with mesenchymal subpopulations has been employed to evaluate the ability of mesenchymal cells to support stem cell regeneration and differentiation. Under these conditions, co-culturing generated organoids called bronchiolar organoids, which contained both Club and ciliated cells. However, due to the absence of basal and goblet cells, these organoid models are mainly used to study the biological characteristics of Club and ciliated cells [85,86]. Furthermore, mouse BASCs have been shown to co-express both bronchiolar and alveolar cell markers. By co-culturing BASCs with lung endothelial cells, researchers successfully generated bronchiolar and bronchioalveolar organoids, providing new model systems for studying lung structure and function [87].

Current airway organoid models have limitations in simulating the 3D structure and physiological features of small airways. Researchers have addressed this by using cell encapsulation techniques, where bronchial epithelial cells from patients are encapsulated in tubular alginate scaffolds, successfully creating a physiologically relevant bronchial organoid model. This model exhibits typical epithelial morphology and tubular structures with visible lumens, closely mimicking the physiological characteristics of the human distal airway [88]. It also facilitates efficient viral infections via intraluminal injection, overcoming challenges seen with traditional infection methods. Previously, organoid construction relied on lung tissue specimens or iPSCs, which were limited by specimen and complex procedures. A recent study reported generating airway organoids from bronchoalveolar lavage fluid (BALF) [89]. After dissociating and filtering BALF cells, they were cultured in airway or alveolar organoid systems, generating stable organoids within 7–10 days, with passaging maintained for at least 6 months. The system also allows for freezing and recovery. Single-cell sequencing analysis revealed that BALF-derived airway organoids were primarily composed of basal cells, with smaller proportions of secretory/goblet cells, ciliated cells, neuroendocrine cells, and tuft cells. This technique offers a new research strategy for modeling lung diseases using accessible human lung samples. However, organoids grown in extracellular matrices, including airway organoids, typically exhibit apical polarity, with the apical surface enclosed within the organoid lumen. To address this, researchers reversed the polarity in airway organoids by removing the extracellular matrix, creating an “apically outward” model [90]. This model, with its apical orientation, offers a more physiologically relevant approach to studying respiratory infections and airway diseases.

### 2.3. Alveolar Organoid

In 2013, Barkauskas and colleagues first demonstrated that AT2 cells are the primary stem cells of the alveolus through both in vivo and in vitro experiments. By placing lineage-labeled AT2 cells in a 3D culture environment, they generated an “alveolar layer” with self-renewing capabilities, which contained AT2 cells and cells expressing AT1 markers [66]. Co-culturing with primary *PDGFRα*-positive lung mesenchymal cells promoted more efficient alveolar layer growth and differentiation [66]. Within this AT2 cell population, Wnt-responsive alveolar epithelial progenitor (AEP) cells play a crucial role in distal lung regeneration after acute injury. These AEPs remain stable during homeostasis but proliferate rapidly post-injury to regenerate much of the alveolar epithelium. Further studies identified *TM4SF1* as a surface marker for human AEP cells, aiding the identification of AEP subpopulations in normal human lungs [91]. Co-culture with human fetal lung fibroblasts enabled these subpopulations to generate functional alveolar layers, offering insights into human alveolar regeneration and disease modeling [91]. Additionally, the team also developed immortalized human AT2 cell lines using SV40 large T antigen lentiviral transfection and a *RHO*/*ROCK* pathway inhibitor (Y-27632), which formed alveolar organoids when co-cultured with mouse lung fibroblasts [25]. Mesenchymal stem cells (MSCs) secreted factors like TSP1 and MMP-9, which regulated lung progenitor cell function and promoted alveolar differentiation in co-culture with lung progenitor organoids [92]. Karen Hoffmann and colleagues [93] identified a subpopulation of *HTII-280^+^*/*EpCAM^+^* alveolar epithelial progenitor cells in adult lungs using single-cell RNA sequencing. These cells, enriched in stem cell markers, generated dual-differentiated bronchioalveolar organoids in 3D culture, with their differentiation direction influenced by *GSK-3β* inhibition (CHIR99021), which promoted alveolar differentiation via *WNT* signaling [93,94,95]. Recently, a method was developed to generate AT2-derived alveolar organoids without fibroblasts [95]. Specifically, by using Matrigel containing Jagged-1 and a specific mixture of medium containing FGF7, Noggin, SB431542, and CHIR99021, alveolar organoids derived from human AT2 cells were successfully established. These advances deepen our understanding of alveolar biology and provide models for studying lung diseases and regenerative processes.

In addition, BASCs have been shown to regenerate both bronchiolar and alveolar epithelia under various injury conditions [61,96]. Endothelial cell-derived *BMP4*/*NFATc1*/*TSP1* signaling enhances BASC proliferation and differentiation into an alveolar phenotype [87]. Co-culturing BASCs with lung endothelial cells forms bronchioalveolar organoids, but these lack AT1 cells in distal alveolar regions [87]. Adding lung mesenchymal cells to the culture generates organoids with mature alveolar-like and proximal airway-like regions. The distal regions express AT1 and AT2 markers (*SFTPC*, *HOPX*), while proximal regions show basal, goblet, and ciliated cell markers (*TP63*, *FOXJ1*, *MUC5AC*). Introducing alveolar macrophages into organoids aids differentiation and maturation, creating a complex niche. This model provides a new platform for studying lung infections, injuries, and repair [97]. Further research also reported that lung alveolar organoids can be derived from basal cells. Salahudeen et al. [98] cultivated organoids from *Krt5^+^* basal cells, and single-cell sequencing revealed that these organoids had proliferating ITGA6+ and ITGB4+ cells, with *TNFRSF12A*^hi cells showing high clonogenic activity. This offers crucial insights into the source and culture optimization of distal lung alveolar organoids.

In recent years, significant progress has been made in the development of alveolar organoids derived from ESCs and iPSCs. Researchers have successfully constructed organoids derived from fetal lung tip (LBT) cells in a culture medium containing FGF-7, FGF-10, CHIR, DCI (dexamethasone, cAMP, and intracellular cAMP activator IBMX), T3 (triiodothyronine), and the *Notch* inhibitor DAPT. Through co-culturing these LBT-derived organoids with freshly isolated human lung mesenchymal cells, differentiation of the organoids towards the alveolar lineage was induced [80]. Furthermore, when tip progenitor cells sourced from fetal lung tissue (from 8.5 to 19 weeks of gestation) were cultured with isolated *RSPO2^+^* mesenchymal cells, *RSPO2* signaling was observed to facilitate the maintenance of the tip and its differentiation towards the alveolar lineage. The enhanced *RSPO2^−^* mediated signaling supports distal (alveolar) differentiation of the tip organoids via *WNT* signaling pathways [81]. Dye et al. first reported human lung organoids (HLOs) from hPSCs, using SAG to activate *HH* signaling, boosting *NKX2.1* expression and forming organoids with both proximal and distal airway structures [99]. These organoids included multiple alveolar cell types, effectively mimicking human lung structure. Later studies used ESCs/iPSCs to create alveolar organoids with diverse epithelial cells like AT1, AT2, and basal cells [100]. Culturing these organoids under controlled oxygen and pressure conditions and using co-cultures has led to complex, multi-cellular structures resembling in vivo alveoli [101]. For example, iPSC-derived alveolar epithelial cells (iAECs) and their co-cultures with macrophages have been used to model the capillary-alveolar barrier [102]. Seo et al. [103] created human iPSC-derived alveolar organoids with macrophages (iMACs) by injecting iMACs into human hiPSC-derived alveolar organoids. These iMACs, which persisted for up to 14 days, showed increased *IL-1β* and *TNF-α* gene expression under LPS-induced inflammation. Lung mesenchyme provides structural support for lung tissue and facilitates differentiation. Yamamoto et al. proposed embedding alveolar organoids in extracellular matrix hydrogels with fetal lung fibroblasts, which supported *SFTPC^+^* alveolar organoid expansion and AT2 stemness [104]. Tamai et al. [105] used *NKX2-1^+^* lung progenitor cells and iPSC-derived mesenchymal cells (iMES) to generate alveolar organoids. iMES, expressing *R-spondin-2* and *R-spondin-3*, promoted differentiation of *SFTPC^+^ EPCAM^+^* AT2 cells, emphasizing epithelial–mesenchymal interactions in lung organogenesis. By optimizing culture medium components like CHIR99021, FGF10, KGF, and DAPT, researchers have successfully established a long-term expansion system for iPSC-derived alveolar organoids [104]. Additionally, customized hyaluronic acid hydrogels with micro-chambers have been used to produce uniform alveolar organoids from human iPSC-derived AT2 cells in a matrix-free system. These ESC/iPSC-derived alveolar organoid models offer valuable platforms for studying lung development, lineage tracing, and cell interactions in disease processes [106].

## 3. Applications of Respiratory Organoid Models to Study Lung Diseases

### 3.1. Pulmonary Fibrosis

Respiratory organoids derived from human ESC/iPSC and ASC can simulate key features of pulmonary fibrosis, including idiopathic pulmonary fibrosis (IPF) and cystic fibrosis (CF) [107]. Although significant breakthroughs have been made in these studies, clinical treatment options for pulmonary fibrosis remain limited. Organoid technology has played an important role in uncovering disease mechanisms and advancing drug development.

#### 3.1.1. Idiopathic Pulmonary Fibrosis

Idiopathic pulmonary fibrosis (IPF) is a chronic interstitial lung disease primarily associated with alveolar epithelial cell injury, aberrant fibroblast activation, and excessive extracellular matrix (ECM) deposition. By using CRISPR/Cas9-based gene editing, TGF-β1, or bleomycin stimulation, researchers have generated IPF-like organoids that display fibroblast proliferation, senescence, and ECM accumulation [108]. For example, Strikoudis et al. developed *HPS1^−/−^* organoids that spontaneously developed fibrosis, while Kim et al. established a TGF-β1–induced hPSC-derived alveolar organoid model for drug testing [108,109]. Chen et al. constructed lung bud organoids (LBOs) from hPSCs and demonstrated that introducing HPS1 mutations produced early-onset fibrotic features [110]. Patient-derived lung spheroids (3D multicellular structures) also recapitulate fibrotic remodeling and show patient-specific responses to antifibrotic drugs, supporting their use as preclinical models [111]. In addition, mutations in *HPS2* or *HPS4* can induce fibrotic changes in LBOs, while *IL-11* knockout attenuates fibrosis in *HPS4^−/−^* organoids, highlighting *IL-11* as a potential therapeutic target [110,112]. Exosomes released from lung spheroid cells, as well as 3D spheroids cultured from hPSCs, have shown regenerative and anti-fibrotic potential, suggesting novel directions for lung tissue repair [113]. To further explore the molecular drivers of IPF, hiPSC-derived alveolar organoids treated with a fibrosis-inducing cytokine mix (TNFα, PDGF-AB, TGF-β, LPA) exhibited decreased surfactant protein C (SPC) and increased vimentin, *KRT17*, and *KRT8* expression, indicating epithelial injury and maladaptive repair [114]. Pathways such as *WNT*, *BMP*, *IL-6*/*STAT3*, and *FGF7* signaling regulate mesenchymal activity and AT2 cell renewal, while impaired glutamine metabolism reduces alveolar regenerative capacity [115]. These findings emphasize the interplay between epithelial dysfunction and mesenchymal activation in fibrosis. Due to the limitations of alveolar epithelial cell in vitro culture and expansion conditions, specific lung fibrosis models targeting alveolar epithelial cells are rare. Takahiro Suezawa et al. [116] developed fibroblast-dependent alveolar organoids from hPSCs and human fetal lung fibroblasts, enabling long-term expansion and drug screening for IPF. In this model, a TGF-β-driven positive feedback loop in senescent AT2 cells accelerated fibroblast activation, providing a mechanistic basis for disease progression. Takahiro Suezawa et al. developed fibroblast-dependent alveolar organoids from hPSCs and human fetal lung fibroblasts, enabling long-term expansion and drug screening for IPF. In this model, a TGF-β-driven positive feedback loop in senescent AT2 cells accelerated fibroblast activation, providing a mechanistic basis for disease progression. hPSC-derived lung spheroids and organoids more closely resemble natural lung tissue in cell distribution and internal structure. However, they remain fetal-like in maturity, which limits their ability to fully model IPF, which involves age-related epithelial senescence and chronic disease remodeling [117].

Unlike lung fibrosis organoids derived from ESC/iPSC, Surolia et al. directly cultured alveolar organoids from the lung tissue of IPF patients, which replicated in vivo fibrotic characteristics [111]. Similarly, Wilkinson et al. developed a human fetal mesenchymal organoid model that exhibited tissue contraction and strong α-SMA and collagen I expression following TGF-β stimulation, recapitulating early fibrotic remodeling [118]. AT2 cells play a crucial role in alveolar injury repair and fibrosis progression. In human and mouse alveolar organoids, *Cebpa* downregulation promoted fibrogenic gene expression, whereas *Cebpa* overexpression restored Sftpc and suppressed fibrosis markers, highlighting its protective role. Consistent with this, activation of the *WNT*/β-catenin pathway via FZD-specific agonists enhances epithelial renewal and alleviates fibrotic phenotypes. Metabolic regulation also contributes to IPF pathogenesis. Glutamine—a key substrate for energy and biosynthesis—is significantly reduced in AT2 cells from IPF patients and bleomycin-treated mice. Inhibition of glutamine metabolism impairs proliferation and differentiation of AT2-derived alveolar organoids, limiting epithelial regeneration [115]. Additionally, IPF lungs show an increase in abnormal basal-like *KRT17^+^* cell populations. Airway basal cells from IPF patients form excessive bronchial spheroids with pro-fibrotic properties, in which *SRC* signaling is enriched. Pharmacologic *SRC* inhibition reduces organoid formation and fibrosis progression in vitro and in vivo [119]. Long-term accumulation of basal cells also helps establish a pro-fibrotic niche, as basal cell-derived WNT7A activates fibroblasts and impairs AT2 renewal [120]. These adult stem cell-derived organoid models offer simpler, faster construction and a more faithful representation of human fibrotic tissue. Co-culture systems combining adult epithelial organoids with fibroblasts have become powerful tools for dissecting epithelial–mesenchymal crosstalk in pulmonary fibrosis. Using primary AT2 cells and lung fibroblasts from mice and humans, researchers demonstrated that bleomycin-induced DNA damage triggers *p53* activation and a self-sustaining *TGF-β* feedback loop within AT2 cells, driving inflammation-independent fibrosis. Targeting this autocrine loop may represent a therapeutic strategy for non-inflammatory fibrotic disease [121]. Further supporting this concept, Sui et al. showed that deletion of ANT1 in primary AT2 cells led to cellular senescence and loss of regenerative capacity, resulting in fewer alveolar organoids [122]. Moreover, in AT2–fibroblast co-culture organoids, activation of the *TGF-β1*/*sFRP2* non-canonical *WNT* signaling axis promoted differentiation into *KRT17^+^* basal-like cells, marking the early stages of IPF pathogenesis [123]. Inhibition of *ROCK1/2* reversed TGF-β–induced fibroblast differentiation and restored organoid growth, suggesting *ROCK* signaling as a potential antifibrotic target [124]. Finally, Tan et al. developed airway organoids using adult bronchial epithelial cells, fibroblasts, and microvascular endothelial cells that formed branched tubular structures resembling late fetal lung stages. Although their long-term viability remains limited, these models represent an important step toward building multicellular airway fibrosis organoids [82].

Organoid techniques using patient-derived cells have improved predictions of IPF treatment responses. For instance, iPSCs from IPF patients replicate the fibrotic phenotype, aiding drug efficacy evaluation. iPSC-derived respiratory organoids treated with pirfenidone showed a 35% reduction in collagen (*p* < 0.01) [125]. In a study of 20 IPF patients, organoids from bronchial biopsies assessed drug response: those with >50% collagen reduction from pirfenidone had an 85% 2-year survival rate, versus 45% for <50% reduction (HR = 0.21, 95% CI: 0.06–0.74, *p* = 0.016) [111]. A study on a 58-year-old male with IPF unresponsive to standard treatment used ASC-derived organoids to screen 500 compounds, identifying a TGF-β inhibitor that reduced ECM deposition by 60% (*p* < 0.001) [126]. Off-label treatment improved his FVC by 25% in 3 months and stabilized his condition for a year. These cases demonstrate the translational potential of organoid models in pediatric fibrotic lung diseases.

#### 3.1.2. Cystic Fibrosis

Cystic fibrosis (CF) is a genetic disorder linked to *CFTR* gene defects [127]. CF organoids have advanced *CFTR* gene research significantly. Patient-derived PSC organoids can mimic the genetic defect’s impacts on lung development. McCauley et al. [128] created CF-specific airway organoids derived from iPSCs, revealing Wnt-driven airway patterning, and developed a “low Wnt” culture protocol to generate organoids from *NKX2-1^+^* progenitor cells. These organoids showed *CFTR*-dependent swelling upon forskolin stimulation (a forskolin-induced swelling test widely used to assess *CFTR*-dependent fluid secretion and predict drug responses in CF patients) [129,130]. Sachs et al. [83] developed airway organoids from CF patient ASCs, replicating core CF features, and exhibited significant swelling after *CFTR* modulation and *TMEM16A* activation. Previous studies have used the FIS test to assess *CFTR* modulator responses in distal airway organoids, but results vary due to limited swelling in well-differentiated spherical structures [68,131,132]. Nasal mucosa organoids, however, are more sensitive in detecting *CFTR* function, making them ideal for personalized CF therapies. In 2020, Liu et al. [132] cultured nasal mucosa organoid models from CF patients and healthy subjects, confirming *CFTR* gene expression and differences in lumen formation. They also introduced an automated *CFTR* activity assay using baseline lumen area measurement to evaluate organoid differentiation [67]. Due to the low throughput of functional *CFTR* assays in previous nasal mucosal organoids studies [131,132,133], there is a need for a scalable FIS detection method using airway organoids from nasal brushing samples. Researchers have improved organoid culture by using 2D differentiated nasal epithelial cell monolayers and optimizing conditions with growth factors like neuregulin-1β and IL-1β to enhance *CFTR* modulator response detection [134]. Validation showed stable detection of genotype-specific *CFTR* modulator responses, including the FDA-approved triple regimen (VX-661/VX-445/VX-770) [135]. The short-circuit current (Isc) remains the gold standard for measuring *CFTR* activity, accurately assessing activity levels and fluid transport effects. Measuring *CFTR* activity via short-circuit current in vitro is complex. To simplify this, the ANDERSON team [136] developed an automated nasal mucosa organoid swelling assay. This method accurately assesses *CFTR* activity and is suitable for drug screening. CF patient-derived organoids have been used to establish biobank samples for studies on *CFTR* repair, including precise gene correction using CRISPR/Cas9 [137]. Nasal mucosa organoids are cost-effective, efficient, and directly relevant to *CFTR* function, making them valuable for basic CF research, drug development, high-throughput screening, and personalized medicine.

### 3.2. Chronic Obstructive Pulmonary Disease

Chronic obstructive pulmonary disease (COPD) is a chronic airway inflammatory disease characterized by persistent respiratory symptoms and irreversible airflow obstruction. Significant risk factors for the development of COPD include exposure to harmful particles and gases, notably cigarette smoke and air pollutants. The development of COPD organoid models is commonly facilitated through exposure to cigarette smoke extract (CSE) [138] or particulate matter 2.5 (PM2.5) [139]. Furthermore, organoids derived from the nasal and bronchial epithelial cells of COPD patients demonstrate disease characteristics akin to those observed in COPD, including goblet cell hyperplasia, elevated *MUC5AC* expression, and a reduction in ciliated cells. The utilization of organoid technology has significantly advanced our understanding of the mechanisms governing the self-renewal, survival, and differentiation of epithelial cells in COPD.

Chan et al. [140] successfully established a COPD organoid model derived from lung adult stem cells of COPD patients. Through single-cell transcriptomic analysis, it was observed that COPD organoids, in comparison to healthy controls, exhibited a reduced presence of basal cells and ciliated cells, alongside an increase in goblet cells. This finding indicates that the model was highly consistent with existing in vitro and in vivo COPD models in terms of phenotype, transcriptome, and functionality. Furthermore, their research showed that COPD organoids exhibited enhanced infection efficiency with *SARS-CoV-2*, rendering them an ideal model for studying host–pathogen interactions. Utilizing organoid models derived from COPD patients, researchers identified an important intermediate cell population involved in the abnormal developmental trajectory of basal cells in COPD patients. Transcriptomic analysis further suggested that critical signaling pathways, including those related to mitochondrial dysfunction, endoplasmic reticulum stress, ciliary remodeling, extracellular matrix remodeling, and pro-inflammatory cytokine signaling, are disrupted in COPD pathogenesis. During the processes of basal cell regeneration and differentiation, cellular metabolism undergoes dynamic regulation. The researchers observed that basal cells preferentially metabolize lactate through glycolysis during early regeneration [141]. In contrast, during lumen formation, there is a metabolic shift towards the tricarboxylic acid (TCA) cycle [141]. Furthermore, they demonstrated that dysfunction in mitochondrial pyruvate metabolism is a crucial mechanism contributing to basal cell hyperplasia, impaired differentiation of ciliated cells, and the accumulation of intermediate cells. Mesenchymal stem cells, known for their multipotency, secrete regenerative growth factors and immunomodulatory/anti-inflammatory factors, including microRNAs [142,143,144,145]. These properties have led to their widespread application in the treatment of various diseases. In animal models of emphysema, Mesenchymal stem cells have been shown to effectively enhance lung function and improve lung tissue structure, while facilitating the repair of distal alveolar damage. Organoids formed by co-culturing alveolar epithelial cells from non-COPD and COPD patients with lung mesenchymal stem cells suggest that mesenchymal stem cells derived from COPD patients can affect the formation capacity of organoids. Co-culture results showed the formation of larger diameter alveolar organoids with lower early-stage AT2 marker expression, indicating impaired self-renewal and differentiation of alveolar progenitor cells [146]. These findings suggest that abnormal mesenchymal stem cell function may be one reason for the failure of lung tissue repair in COPD.

Key molecular signaling pathways, including *Notch*, *TGF-β*, and *WNT* signaling, play a crucial role in the differentiation of AT2 cells into AT1 cells and in the repair of alveolar epithelium. Disruption of the *WNT*/*β-catenin* pathway can lead to dysregulation of distal alveolar epithelial progenitor cell homeostasis and the development of emphysema. Smoking has been shown to downregulate the expression of the *WNT* receptor protein FZD in both human and murine alveolar epithelial cells, thereby inhibiting *WNT*/*β-catenin* signaling activation and impairing the regenerative repair function of COPD organoids [147]. In studies involving murine respiratory organoids exposed to particulate matter (PM2.5), researchers have demonstrated that impaired AT2 to AT1 cell transition may be a mechanism by which PM2.5 contributes to COPD pathogenesis [139]. Given the structural differences between human and mouse airways, in vivo animal models are limited in their ability to fully replicate human airway architecture. Consequently, researchers have employed human respiratory organoid models to identify a unique secretory cell lineage in human distal lung tissue, referred to as RAS cells. RAS cells have the capacity to differentiate into human AT2 cells via *Notch* and *WNT* signaling pathways, thereby contributing to alveolar regeneration. However, chronic smoke exposure induces abnormal differentiation of RAS cells into AT2 cells, with this abnormal RAS-AT2 differentiation trajectory potentially playing a crucial role in the pathogenesis of COPD [148].

Furthermore, organoid technology also holds significant advantages in drug screening applications. In a study by Wu et al. [138] transcriptomic data from COPD patients and COPD mouse models exposed to CSE were utilized to identify and investigate potential druggable gene targets for COPD, including the E-series prostaglandin receptor (*EP*) and prostacyclin receptor (*IP*). In their study, the researchers developed mouse and human airway and alveolar organoids by co-culturing mouse *CD31^−^*/*CD45^−^*/*Epcam^+^* lung epithelial cells with CCL-206 mouse lung fibroblasts, and human *CD31^−^*/*CD45^−^*/*Epcam^+^* lung epithelial cells with MRC5 human lung fibroblasts. These organoids were subsequently exposed to different concentrations of CSE. The findings revealed that alveolar organoids exhibited significantly greater sensitivity to cigarette smoke compared to airway organoids, with CSE exposure markedly inhibiting the formation of alveolar organoids. Moreover, the organoids were utilized for potential drug target screening, demonstrating that EP4 and IP agonists exerted beneficial therapeutic effects on distal alveolar organoids compromised by CSE exposure. Additionally, Rita Costa et al. screened effective *WNT*/β-catenin activators such as amlexano using mouse COPD organoids and in vivo animal models, proving that amlexano could be a novel drug for improving COPD emphysema [149].

### 3.3. Respiratory Cancers

#### 3.3.1. Lung Cancer

Research suggests that cancer may originate from endogenous stem cells, within which a subset of stem cells, termed “cancer stem cells” (CSCs), is believed to regulate tumor growth and tumorigenesis [150]. Lung cancer is the most common type of cancer and the leading cause of cancer-related death among men. This type of cancer encompasses a variety of subtypes, each characterized by highly diverse mutation patterns, which complicates both research and treatment efforts. According to the CSCs theory, lung cancer is conceptualized as a stem cell disease resulting from the malignant transformation of adult lung stem cells [151]. Currently, the in vitro disease models employed in cancer research predominantly include 2D cell culture cancer cell lines, patient-derived tumor xenografts (PDXs), and tumor organoids (patient-derived organoids, PDOs). Due to their ease of manipulation and cost-effectiveness, the growth of cancer cell lines in 2D cultures limits their ability to accurately represent the heterogeneity inherent in lung cancer. PDXs are developed by implanting patient tumors into immunodeficient mice, thereby providing a substantial degree of structural fidelity to the original cancer. Due to high costs, lengthy modeling periods, and low success rates, PDXs have limitations. In contrast, PDOs, cultured from patient cancer tissues, offer advantages like higher success rates, shorter modeling times, and lower costs.

Organoid systems derived from cancer tissues have been applied to the primary lung cancer cell cultures [152]. Zhang et al. successfully established patient-derived non-small cell lung cancer (NSCLC) tumor spheres [153]. These tumor spheres contained cancer cells with a high nuclear-to-cytoplasm ratio, deep-staining nuclei, and irregular nuclear membranes, with adenocarcinoma marker *Thyroid Transcription Factor-1* (*TTF-1*) staining positive, consistent with the patient’s original tumor sample, further confirming their adenocarcinoma origin [153]. Serial passage experiments showed that the cytological characteristics of these tumor spheres remained stable, and clinical markers were consistently retained throughout the passage process [153]. Research conducted by Weeber et al. [154] and Sato et al. [18] has corroborated that tumor organoids, aside from their reduced construction times, can maintain genetic and morphological stability even after long-term expansion. These findings provide reliable evidence for the application of tumor organoids. Furthermore, by manipulating organoids derived from normal tissues, it is possible to simulate lung cancer. For instance, Park et al. generated organoids from human bronchial epithelial cells and employed gene editing techniques to upregulate the medulloblastoma and B-cell lymphoma genes [155]. These organoids were subsequently transplanted into immunosuppressed mice, resulting in tumors that closely resembled small cell lung cancer (SCLC). Additionally, Kim et al. [156] developed a basic culture medium devoid of Wnt3a and Noggin to inhibit normal cell growth, thereby creating a library of 80 lung cancer organoid samples encompassing five subtypes of lung cancer: adenocarcinoma, squamous cell carcinoma, small cell carcinoma, adenosquamous carcinoma, and large cell carcinoma. These lung cancer organoids (LCOs) preserved the genetic traits and tissue structure of the original cancer, expressing specific tumor markers and maintaining stable morphology in vitro for up to six months. Using patient samples, primarily epithelial cells, the success rates for culturing were highest for 2D primary lung cancer cells (100%), followed by LCOs (87%), and PDXs (3%). LCOs required only 4 weeks to cultivate, compared to 3–6 months for PDXs, and closely resembled patient tissue morphology. These data demonstrate that LCOs have outstanding advantages in terms of modeling success rate, time, and cost. Additionally, LCOs successfully generated PDXs when transplanted into immunodeficient mice. Loss of liver kinase B1 (*LKB1*) function is common in adenocarcinoma. Zhang et al. showed that deleting *LKB1* in *KRAS*-positive organoids can convert *KRAS*-positive adenocarcinomas into squamous cell carcinoma (SCC), a process confirmed in both spontaneous and transplant models [157,158]. These findings suggest new lung cancer treatment strategies, with targeted therapies for *LKB1* loss showing promise in preclinical studies. Additionally, organoids from *Bcl11a* knockout mice confirmed *BCL11A* as an oncogene in SCC [159].

Beyond their advantages in lung cancer modeling and mechanistic research, respiratory organoids also hold substantial promise in the realm of lung cancer drug discovery and screening. Traditional 2D cell culture systems have often led to the identification of anticancer drugs that exhibit limited efficacy in clinical trials [160]. In contrast, patient-derived organoids, which retain the specific functions of the primary tumor, offer a robust platform for high-throughput drug screening and serve as an important tool for assessing the efficacy of personalized treatments [83]. For example, Barrera-Rodríguez et al. developed a multicellular tumor spheroid model to investigate alterations in drug sensitivity and the molecular mechanisms of multicellular drug resistance [161]. Hai et al. [162] used CRISPR/Cas9 technology to perform gene editing on mouse-derived organoids, successfully knocking out several tumor suppressor genes. By comparing these organoids with patient-derived cell lines, researchers found them to closely resemble human lung squamous cell carcinoma genetically and phenotypically. They proposed a combination therapy hypothesis using immune checkpoint blockade and DNA damage-inducing treatments. Organoids serve as an ideal model for assessing patient-specific drug responses, reacting to drugs based on genomic changes, and providing insights into drug resistance mechanisms. For example, lung cancer organoids with the *BRCA2* p.W2619C mutation were more sensitive to olaparib than those with the *BRCA2* p.M965I mutation, with olaparib disrupting the structure of the former and inhibiting PDX tumor growth [156]. Wang et al. successfully constructed a patient-derived organoid model from an advanced adenocarcinoma harboring the *HER2*-A775_G776YVMA insertion mutation [163]. Utilizing this organoid model, the researchers evaluated the efficacy of *HER2*-targeted therapeutics (afatinib, T-DM1, and pyrotinib). Their findings indicated that pyrotinib exhibited the most significant tumor suppression effects at clinically relevant concentrations both in vivo and in vitro. Furthermore, the researchers assessed the effectiveness of neratinib, afatinib, and gefitinib, proposing that the combination of neratinib and trastuzumab should be considered for clinical trials targeting *HER2*-mutant lung cancer. Additionally, by combining patient-derived organoids with high-throughput screening systems, the researchers examined the efficacy of *EGFR* and *HER2* inhibitors [164]. In summary, these organoid-based drug screening systems provide important evidence for personalized cancer treatment strategies.

Patient-derived lung cancer organoids exhibit a high fidelity in replicating the tumor’s immune microenvironment. Through the co-culture of lung cancer organoids, autologous peripheral blood lymphocytes in a medium supplemented with interferon-γ, IL-2, and anti-PD-1 antibodies, there is a significant promotion of the expansion and enrichment of tumor-reactive CD4^+^T cells and CD8^+^T cells [165,166]. This methodology maintains the integrity of the tumor’s T cell receptor repertoire and effectively emulates the mechanism of action of immune checkpoint inhibitors [167], thereby facilitating the advancement and implementation of T cell therapies in the treatment of lung cancer.

#### 3.3.2. Nasopharyngeal Carcinoma

Nasopharyngeal carcinoma (NPC) starts in nasopharyngeal epithelial cells. Common NPC cell lines often suffer from HeLa cell contamination, lack of molecular diversity, and loss of the *Epstein–Barr virus* (*EBV*) genome over time. Respiratory organoids are increasingly being recognized as a promising model for NPC research. The research team led by Li Gang successfully developed the inaugural patient-derived nasopharyngeal carcinoma organoid (NPCO) model and biobank [73]. Compared to NPCOs, organoids derived from normal nasal mucosa exhibit three morphological types: regular vesicles, irregular vesicles, and irregular liquid shapes, whereas NPCOs consistently manifest a regular spherical form. The study further revealed that recurrent NPCOs exhibit a higher proportion of *Bmi-1*-positive cells compared to primary NPCOs, suggesting that *Bmi-1* expression may play a role in NPCO generation. These nasopharyngeal cancer organoids effectively preserve the pathological and physiological characteristics and genetic heterogeneity of the original tumor. Consequently, they hold significant potential for advancing the development of novel anticancer drugs, investigating the mechanisms underlying NPC development, recurrence, metastasis, and resistance, and formulating personalized treatment strategies.

## 4. Conclusions and Future Direction

Organoids, resembling real tissues and organs, are ideal in vitro models for research. This review outlines methods for creating respiratory organoid models and recent progress in lung disease research. Their use has been crucial for understanding lung epithelial stem/progenitor cell changes in disease, advancing personalized medicine, and exploring lung regeneration. However, current organoid systems still have limitations in replicating in vivo cell maturity, interactions, and complexity.

Currently, organoid cultivation primarily relies on mouse-derived extracellular matrix substitutes like Matrigel, which can vary in terms of reproducibility during research. These substitutes may also contain unknown pathogens, limiting their direct application in clinical transplantation. Clinical-grade collagen could address these issues [168], and bioengineering strategies may further optimize cultivation techniques [169].

A key challenge for using organoid culture methods in regenerative medicine is their inability to mimic multi-organ pathology due to missing cell lineages from different organs. Advanced cellular engineering, such as co-culture of respiratory organoids with niche cells or other organoids, is crucial to address this. Progress is being made with multi-lineage organoids; for example, merging organoids from various brain regions of hPSCs has allowed researchers to replicate neuronal migration, projection, and neurodevelopmental disorders, enhancing the study of multicellular interactions [170,171]. These “assemblies” are multi-regional organoids combining various cell lineages to model complex liver and gallbladder diseases [172,173]. Advances in sequencing have allowed the integration of previously unknown lung cell types, such as *CFTR*-rich ionocytes, into organoids, crucial for understanding cystic fibrosis [174]. Using ALI technology, researchers co-cultured neuroendocrine cells with fetal lung fibroblasts to create respiratory organoids with rare neuroendocrine cells [175], closely resembling natural lung epithelial cells. Advanced techniques like single-cell RNA sequencing and spatial transcriptomics validate these organoids, enhancing disease modeling and paving the way for future multi-organ organoids and regenerative medicine applications. Current tumor organoids lack essential elements like biomechanical stimulation, matrix components, blood vessels, and immune cells [176], limiting their use in cancer research. Integrating these could enhance organoid systems, offering better solutions for cancer research and treatment. Recent strategies involve organ-on-a-chip technology to simulate multi-organ interactions and 3D bioprinting with hydrogel to create complex respiratory organoids [177,178]. These innovations pave the way for precise cancer research and personalized treatment.

Tissue maturity is a third key limitation of organoid technology in research and clinical applications. PSC-derived organoids often lack cellular maturity, hindering accurate simulation of human organs. Their fetal-like characteristics can lead to discrepancies in replicating adult disease pathophysiology, as they may not fully capture the complex tissue structure and function of adult lungs. In conditions like IPF and COPD, age-related changes such as fibrosis and cellular senescence are crucial but less evident in immature organoids from ESCs or iPSCs. Thus, while useful for studying early disease mechanisms, these models require cautious interpretation for adult-onset diseases. To address this, researchers use strategies like pre-treating organoids with small molecules such as BDNF (brain-derived neurotrophic factor) to speed up maturation [179]. Additionally, enhancing oxygenation and nutrient diffusion, particularly through vascularization, improves tissue maturity and extends organoid survival [3]. A study demonstrated the coordinated development of human heart-lung development by inducing multi-lineage differentiation of hiPSCs, highlighting the cardiac lineage’s role in alveolar maturation [180]. Wimmer et al. developed a method to transform hiPSCs into mesodermal cells and then into vascular organoids using growth factors like VEGF-A and FGF2 [23]. When transplantation into murine models, these organoids successfully developed a complete vascular system, thereby providing a valuable model for investigating diabetic vascular complications and facilitating the development of therapeutic interventions. Moreover, researchers have vascularized human respiratory organoids derived from hiPSC lung progenitor cells and endothelial cells, demonstrating differentiation into AT2 and AT1 cells, as well as eliciting inflammatory responses [181]. In a pioneering study, scientists have, for the first time, developed highly vascularized lung and intestinal organoids from human iPSCs [181]. This was accomplished by engineering a 3D system that mimics human embryonic development, allowing for the co-differentiation of mesoderm and endoderm to form blood vessels and lung/gut epithelium within a single spheroid. This achievement overcomes the limitations of traditional organoid models, which typically lack functional vasculature and organ-specific stroma, thereby providing a novel platform for the study of organ development, disease pathogenesis, and regenerative medicine. Our recent study reveals that *BMPR2* mutation impairs late-stage lung progenitor differentiation and airway epithelial maturation. *BMPR2*-mutant airway organoids exhibit impaired goblet and ciliated cell development, with increased basal/club cell markers, suggesting arrested differentiation [182]. These insights highlight *BMPR2*’s role in lung development and its potential link to pulmonary hypertension, offering valuable perspectives for understanding lung development, inflammatory lung injuries, and advancing organoid technology.

In addition, limited reproducibility is a major challenge in organoid research, hindering the creation and control of advanced organoids. Key factors affecting the reproducibility of organoids include batch differences, production scalability, cell composition stability, and structural complexity [3]. Developing automated culture and analysis platforms is crucial to address these issues, enabling standardized large-scale organoid production and high-throughput drug screening [183]. While mouse respiratory organoids show high complexity, identifying equivalent human cells could lead to more complex, personalized organoids from patient samples. This approach will enhance model accuracy for studying human lung diseases. Future use of integrated organoid chip-based culture platforms is anticipated to improve precise modeling of chronic lung diseases. Coupled with high-resolution analytical technologies, these platforms will expand opportunities to explore complex pulmonary pathophysiological mechanisms.

Respiratory organoid technology is widely used in biomedical research, drug screening, and gene therapy. Although its application is still being explored, resolving current challenges will improve its relevance to the human respiratory system. The use of AI and machine learning can enhance image analysis and predict drug responses. Integrating single-cell RNA sequencing and spatial transcriptomics offers perspectives for achieving high-resolution characterization of organoid heterogeneity, lineage trajectories, and disease-associated cell states. Combining organoids with microfluidic platforms and organ-on-chip systems can better simulate physiological conditions. These advancements will deepen our understanding of chronic lung disease and improve patient outcomes.

## Figures and Tables

**Table 1:** Difference between ESC/iPSC and ASC-derived organoids.

	ESC/iPSC-Derived Organoids	ASC-Derived Organoids	References
**Potential for differentiation**	Multidirectional differentiation.	Limited ability to differentiate into organoids of specific tissue origin.	[12,13]
**Cellular components**	Complex cellular components, including mesenchymal, epithelial, and even endothelial components.	Single cellular component, mainly epithelial.	[1,14,15]
**Cultivation conditions**	Complex procedure; the procedure involves adding specific growth factor cocktails at each stage to form germ layers (endoderm, mesoderm, ectoderm) and incubating with growth factors, signaling molecules, and cytokines for cell differentiation and maturation.	Can be directly obtained from regenerative human adult tissues using a simpler procedure, but prior knowledge of culturing media from various tissues is required.	[3,15,16,17,18]
**Incubation time**	Generally take several months.	Generally shorter, about 3–4 weeks.	[15,16,17]
**Technical maturity**	The gene knockout and precise gene-editing techniques are better established.	The technology is relatively mature.	[19]
**Maturity of organoids**	Low maturity, most still have a fetal or neonatal component.	High maturity, closer to the adult tissue.	[16,18]
**Self-renewal capacity**	Exhibit a high proliferative capacity; however, they typically lose their potential for further expansion upon reaching the stage of terminal differentiation, ultimately failing to achieve full maturation.	Limited self-renewal capacity for a prolonged period of time.	[16,20]
**Genetic stability**	High genetic stability, but problems can occur with long-term cultures.	High genetic stability maintained during long-term expansion.	[9]
**Other**	Ability to induce neural lineage differentiation and reconstruct neural tube structures in vitro.	ASCs with stemness cannot be obtained from tissues such as the brain, heart, or pancreatic islets.	[15,21,22]
**Application**	ESC/iPSC-derived organoids are typically naïve and resemble fetal tissues, making them excellent models for studying early human organ development.	ASC-derived organoids offer a comprehensive view of adult tissue repair and viral infections, making them ideal for regenerative medicine and disease modeling.	[3,11,23,24]

## Data Availability

No new data were created or analyzed in this study.

## Abbreviations

2D	Two-dimensional
3D	Three-dimensional
GEMMs	Genetically engineered mouse models
PDXs	Patient-derived xenografts
ASCs	Adult stem cells
ESCs	Embryonic stem cells
iPSCs	Induced pluripotent stem cells
AT1	Type I alveolar epithelial
AT2	Type II alveolar epithelial
ALI	Air-liquid interface
IPF	Idiopathic pulmonary fibrosis
CF	Cystic fibrosis
COPD	Chronic obstructive pulmonary disease
NPC	Nasopharyngeal carcinoma

## References

[1] Clevers H. (2016). Modeling Development and Disease with Organoids. Cell.

[2] Zhao Z., Chen X., Dowbaj A.M., Sljukic A., Bratlie K., Lin L., Fong E.L.S., Balachander G.M., Chen Z., Soragni A. (2022). Organoids. Nature reviews. Methods Primers.

[3] Tang X.-Y., Wu S., Wang D., Chu C., Hong Y., Tao M., Hu H., Xu M., Guo X., Liu Y. (2022). Human organoids in basic research and clinical applications. Signal Transduct. Target. Ther..

[4] Saraswathibhatla A., Indana D., Chaudhuri O. (2023). Cell-extracellular matrix mechanotransduction in 3D. Nat. Rev. Mol. Cell Biol..

[5] Baker B.M., Chen C.S. (2012). Deconstructing the third dimension: How 3D culture microenvironments alter cellular cues. J. Cell Sci..

[6] Scalise M., Marino F., Salerno L., Cianflone E., Molinaro C., Salerno N., De Angelis A., Viglietto G., Urbanek K., Torella D. (2021). From Spheroids to Organoids: The Next Generation of Model Systems of Human Cardiac Regeneration in a Dish. Int. J. Mol. Sci..

[7] Bresnahan E., Ramadori P., Heikenwalder M., Zender L., Lujambio A. (2020). Novel patient-derived preclinical models of liver cancer. J. Hepatol..

[8] Corrò C., Novellasdemunt L., Li V.S.W. (2020). A brief history of organoids. Am. J. Physiol. Cell Physiol..

[9] Brevini T., Tysoe O.C., Sampaziotis F. (2020). Tissue engineering of the biliary tract and modelling of cholestatic disorders. J. Hepatol..

[10] Wood L.D., Ewald A.J. (2021). Organoids in cancer research: A review for pathologist-scientists. J. Pathol..

[11] Yang S., Hu H., Kung H., Zou R., Dai Y., Hu Y., Wang T., Lv T., Yu J., Li F. (2023). Organoids: The current status and biomedical applications. Medcomm.

[12] Thomson J.A., Itskovitz-Eldor J., Shapiro S.S., Waknitz M.A., Swiergiel J.J., Marshall V.S., Jones J.M. (1998). Embryonic Stem Cell Lines Derived from Human Blastocysts. Science.

[13] Takahashi K., Yamanaka S. (2006). Induction of pluripotent stem cells from mouse embryonic and adult fibroblast cultures by defined factors. Cell.

[14] McCracken K.W., Aihara E., Martin B., Crawford C.M., Broda T., Treguier J., Zhang X., Shannon J.M., Montrose M.H., Wells J.M. (2017). Wnt/β-catenin promotes gastric fundus specification in mice and humans. Nature.

[15] Brassard J.A., Lutolf M.P. (2019). Engineering Stem Cell Self-organization to Build Better Organoids. Cell Stem Cell.

[16] Schutgens F., Clevers H. (2020). Human Organoids: Tools for Understanding Biology and Treating Diseases. Annu. Rev. Pathol..

[17] Günther C., Winner B., Neurath M.F., Stappenbeck T.S. (2022). Organoids in gastrointestinal diseases: From experimental models to clinical translation. Gut.

[18] Sato T., Stange D.E., Ferrante M., Vries R.G., van Es J.H., Brink S.v.D., van Houdt W.J., Pronk A., van Gorp J., Siersema P.D. (2011). Long-term Expansion of Epithelial Organoids from Human Colon, Adenoma, Adenocarcinoma, and Barrett’s Epithelium. Gastroenterology.

[19] Giani A.M., Chen S. (2021). Human pluripotent stem cell-based organoids and cell platforms for modelling *SARS-CoV-2* infection and drug discovery. Stem Cell Res..

[20] McCracken K.W., Catá E.M., Crawford C.M., Sinagoga K.L., Schumacher M., Rockich B.E., Tsai Y.-H., Mayhew C.N., Spence J.R., Zavros Y. (2014). Modelling human development and disease in pluripotent stem-cell-derived gastric organoids. Nature.

[21] Zhang S.-C., Wernig M., Duncan I.D., Brüstle O., Thomson J.A. (2001). In vitro differentiation of transplantable neural precursors from human embryonic stem cells. Nat. Biotechnol..

[22] Liu Y., Liu H., Sauvey C., Yao L., Zarnowska E.D., Zhang S.-C. (2013). Directed differentiation of forebrain GABA interneurons from human pluripotent stem cells. Nat. Protoc..

[23] Wimmer R.A., Leopoldi A., Aichinger M., Wick N., Hantusch B., Novatchkova M., Taubenschmid J., Hämmerle M., Esk C., Bagley J.A. (2019). Human blood vessel organoids as a model of diabetic vasculopathy. Nature.

[24] Kim H., Park H.J., Choi H., Chang Y., Park H., Shin J., Kim J., Lengner C.J., Lee Y.K., Kim J. (2019). Modeling G2019S-LRRK2 Sporadic Parkinson’s Disease in 3D Midbrain Organoids. Stem Cell Rep..

[25] Tran E., Shi T., Li X., Chowdhury A.Y., Jiang D., Liu Y., Wang H., Yan C., Wallace W.D., Lu R. (2022). Development of human alveolar epithelial cell models to study distal lung biology and disease. iScience.

[26] Kalender M., Bulbul M.V., Kolbasi B., Keskin I. (2022). In 2D and 3D Cell Culture Models, Effects of Endothelial Cells on E-cadherin/β-catenin Expression Levels and Spheroid Sizes in Ishikawa Cells. Asian Pac. J. Cancer Prev..

[27] Laube M., Pietsch S., Pannicke T., Thome U.H., Fabian C. (2021). Development and Functional Characterization of Fetal Lung Organoids. Front. Med..

[28] Chen S., Schoen J. (2019). Air-liquid interface cell culture: From airway epithelium to the female reproductive tract. Reprod. Domest. Anim..

[29] Lacroix G., Koch W., Ritter D., Gutleb A.C., Larsen S.T., Loret T., Zanetti F., Constant S., Chortarea S., Rothen-Rutishauser B. (2018). Air–Liquid Interface In Vitro Models for Respiratory Toxicology Research: Consensus Workshop and Recommendations. Appl. Vitr. Toxicol..

[30] Zhou J., Li C., Sachs N., Chiu M.C., Wong B.H.-Y., Chu H., Poon V.K.-M., Wang D., Zhao X., Wen L. (2018). Differentiated human airway organoids to assess infectivity of emerging influenza virus. Proc. Natl. Acad. Sci. USA.

[31] Kühl L., Graichen P., von Daacke N., Mende A., Wygrecka M., Potaczek D.P., Miethe S., Garn H. (2023). Human Lung Organoids—A Novel Experimental and Precision Medicine Approach. Cells.

[32] Joo H., Min S., Cho S. (2024). Advanced lung organoids for respiratory system and pulmonary disease modeling. J. Tissue Eng..

[33] Velasco S., Kedaigle A.J., Simmons S.K., Nash A., Rocha M., Quadrato G., Paulsen B., Nguyen L., Adiconis X., Regev A. (2019). Individual brain organoids reproducibly form cell diversity of the human cerebral cortex. Nature.

[34] Man W.H., de Steenhuijsen Piters W.A.A., Bogaert D. (2017). The microbiota of the respiratory tract: Gatekeeper to respiratory health. Nat. Rev. Microbiol..

[35] Weibel E.R. (2017). Lung morphometry: The link between structure and function. Cell Tissue Res..

[36] Hewitt R.J., Lloyd C.M. (2021). Regulation of immune responses by the airway epithelial cell landscape. Nat. Rev. Immunol..

[37] Widdicombe J.H., Wine J.J. (2015). Airway Gland Structure and Function. Physiol. Rev..

[38] Rogers T.D., Button B., Kelada S.N.P., Ostrowski L.E., Livraghi-Butrico A., Gutay M.I., Esther C.R., Grubb B.R. (2022). Regional Differences in Mucociliary Clearance in the Upper and Lower Airways. Front. Physiol..

[39] Okuda K., Dang H., Kobayashi Y., Carraro G., Nakano S., Chen G., Kato T., Asakura T., Gilmore R.C., Morton L.C. (2021). Secretory Cells Dominate Airway CFTR Expression and Function in Human Airway Superficial Epithelia. Am. J. Respir. Crit. Care Med..

[40] Gohy S.T., Hupin C., Pilette C., Ladjemi M.Z. (2016). Chronic inflammatory airway diseases: The central role of the epithelium revisited. Clin. Exp. Allergy.

[41] Knudsen L., Ochs M. (2018). The micromechanics of lung alveoli: Structure and function of surfactant and tissue components. Histochem. Cell Biol..

[42] Bluhmki T., Traub S., Müller A.-K., Bitzer S., Schruf E., Bammert M.-T., Leist M., Gantner F., Garnett J.P., Heilker R. (2021). Functional human iPSC-derived alveolar-like cells cultured in a miniaturized 96-Transwell air–liquid interface model. Sci. Rep..

[43] Rindler T.N., Stockman C.A., Filuta A.L., Brown K.M., Snowball J.M., Zhou W., Veldhuizen R., Zink E.M., Dautel S.E., Clair G. (2017). Alveolar injury and regeneration following deletion of ABCA3. J. Clin. Investig..

[44] Evans K.V., Lee J. (2020). Alveolar wars: The rise of in vitro models to understand human lung alveolar maintenance, regeneration, and disease. Stem Cells Transl. Med..

[45] Jain K.G., Zhao R., Liu Y., Guo X., Yi G., Ji H.-L. (2022). Wnt5a/β-catenin axis is involved in the downregulation of AT2 lineage by PAI-1. Am. J. Physiol. Cell. Mol. Physiol..

[46] Strunz M., Simon L.M., Ansari M., Kathiriya J.J., Angelidis I., Mayr C.H., Tsidiridis G., Lange M., Mattner L.F., Yee M. (2020). Alveolar regeneration through a Krt8+ transitional stem cell state that persists in human lung fibrosis. Nat. Commun..

[47] Basil M.C., Katzen J., Engler A.E., Guo M., Herriges M.J., Kathiriya J.J., Windmueller R., Ysasi A.B., Zacharias W.J., Chapman H.A. (2020). The Cellular and Physiological Basis for Lung Repair and Regeneration: Past, Present, and Future. Cell Stem Cell.

[48] Konkimalla A., Tata A., Tata P.R. (2022). Lung Regeneration: Cells, Models, and Mechanisms. Cold Spring Harb. Perspect. Biol..

[49] Hogan B.L., Barkauskas C.E., Chapman H.A., Epstein J.A., Jain R., Hsia C.C., Niklason L., Calle E., Le A., Randell S.H. (2014). Repair and Regeneration of the Respiratory System: Complexity, Plasticity, and Mechanisms of Lung Stem Cell Function. Cell Stem Cell.

[50] Hong K.U., Reynolds S.D., Watkins S., Fuchs E., Stripp B.R. (2004). Basal Cells Are a Multipotent Progenitor Capable of Renewing the Bronchial Epithelium. Am. J. Pathol..

[51] Boers J.E., Ambergen A.W., Thunnissen F.B. (1998). Number and proliferation of basal and parabasal cells in normal human airway epithelium. Am. J. Respir. Crit. Care Med..

[52] Rokicki W., Rokicki M., Wojtacha J., Dżeljijli A. (2016). The role and importance of club cells (Clara cells) in the pathogenesis of some respiratory diseases. Pol. J. Cardio-Thoracic Surg..

[53] Van Keymeulen A., Blanpain C. (2012). Tracing epithelial stem cells during development, homeostasis, and repair. J. Cell Biol..

[54] Rock J.R., Onaitis M.W., Rawlins E.L., Lu Y., Clark C.P., Xue Y., Randell S.H., Hogan B.L.M. (2009). Basal cells as stem cells of the mouse trachea and human airway epithelium. Proc. Natl. Acad. Sci. USA.

[55] Tata P.R., Mou H., Pardo-Saganta A., Zhao R., Prabhu M., Law B.M., Vinarsky V., Cho J.L., Breton S., Sahay A. (2013). Dedifferentiation of committed epithelial cells into stem cells in vivo. Nature.

[56] Chen H., Matsumoto K., Brockway B.L., Rackley C.R., Liang J., Lee J.-H., Jiang D., Noble P.W., Randell S.H., Kim C.F. (2012). Airway Epithelial Progenitors Are Region Specific and Show Differential Responses to Bleomycin-Induced Lung Injury. Stem Cells.

[57] Rawlins E.L., Okubo T., Xue Y., Brass D.M., Auten R.L., Hasegawa H., Wang F., Hogan B.L. (2009). The Role of Scgb1a1+ Clara Cells in the Long-Term Maintenance and Repair of Lung Airway, but Not Alveolar, Epithelium. Cell Stem Cell.

[58] Butler C.R., Hynds R.E., Gowers K.H.C., Lee D.D.H., Brown J.M., Crowley C., Teixeira V.H., Smith C.M., Urbani L., Hamilton N.J. (2016). Rapid Expansion of Human Epithelial Stem Cells Suitable for Airway Tissue Engineering. Am. J. Respir. Crit. Care Med..

[59] Danahay H., Pessotti A.D., Coote J., Montgomery B.E., Xia D., Wilson A., Yang H., Wang Z., Bevan L., Thomas C. (2015). Notch2 Is Required for Inflammatory Cytokine-Driven Goblet Cell Metaplasia in the Lung. Cell Rep..

[60] Hild M., Jaffe A.B. (2016). Production of 3-D Airway Organoids from Primary Human Airway Basal Cells and Their Use in High-Throughput Screening. Curr. Protoc. Stem Cell Biol..

[61] Kim C.F.B., Jackson E.L., Woolfenden A.E., Lawrence S., Babar I., Vogel S., Crowley D., Bronson R.T., Jacks T. (2005). Identification of Bronchioalveolar Stem Cells in Normal Lung and Lung Cancer. Cell.

[62] Salwig I., Spitznagel B., Vazquez-Armendariz A.I., Khalooghi K., Guenther S., Herold S., Szibor M., Braun T. (2019). Bronchioalveolar stem cells are a main source for regeneration of distal lung epithelia in vivo. EMBO J..

[63] Kotton D.N., Morrisey E.E. (2014). Lung regeneration: Mechanisms, applications and emerging stem cell populations. Nat. Med..

[64] Song H., Yao E., Lin C., Gacayan R., Chen M.-H., Chuang P.-T. (2012). Functional characterization of pulmonary neuroendocrine cells in lung development, injury, and tumorigenesis. Proc. Natl. Acad. Sci. USA.

[65] Li K., Li M., Li W., Yu H., Sun X., Zhang Q., Li Y., Li X., Li Y., Abel E.D. (2019). Airway epithelial regeneration requires autophagy and glucose metabolism. Cell Death Dis..

[66] Barkauskas C.E., Cronce M.J., Rackley C.R., Bowie E.J., Keene D.R., Stripp B.R., Randell S.H., Noble P.W., Hogan B.L. (2013). Type 2 alveolar cells are stem cells in adult lung. J. Clin. Investig..

[67] Liu Z., Anderson J.D., Natt J., Guimbellot J.S. (2021). Culture and Imaging of Human Nasal Epithelial Organoids. J. Vis. Exp. JoVE.

[68] Brewington J.J., Filbrandt E.T., LaRosa F., Ostmann A.J., Strecker L.M., Szczesniak R.D., Clancy J.P. (2018). Detection of CFTR function and modulation in primary human nasal cell spheroids. J. Cyst. Fibros..

[69] Gamage A.M., Tan K.S., Chan W.O.Y., Liu J., Tan C.W., Ong Y.K., Thong M., Andiappan A.K., Anderson D.E., Wang Y. (2020). Infection of human Nasal Epithelial Cells with SARS-CoV-2 and a 382-nt deletion isolate lacking ORF8 reveals similar viral kinetics and host transcriptional profiles. PLoS Pathog..

[70] Chiu M.C., Li C., Liu X., Song W., Wan Z., Yu Y., Huang J., Xiao D., Chu H., Cai J.-P. (2022). Human Nasal Organoids Model SARS-CoV-2 Upper Respiratory Infection and Recapitulate the Differential Infectivity of Emerging Variants. mBio.

[71] Rajan A., Weaver A.M., Aloisio G.M., Jelinski J., Johnson H.L., Venable S.F., McBride T., Aideyan L., Piedra F.-A., Ye X. (2022). The Human Nose Organoid Respiratory Virus Model: An Ex Vivo Human Challenge Model To Study Respiratory Syncytial Virus (RSV) and Severe Acute Respiratory Syndrome Coronavirus 2 (SARS-CoV-2) Pathogenesis and Evaluate Therapeutics. mBio.

[72] Wang K., Yu Y., Han R., Wang X., Zhao Y., Tang H., Li G. (2022). Establishment of a culture system for human nasal mucosa organoids with controllable differentiation. Nan Fang Yi Ke Da Xue Xue Bao.

[73] Wang X.-W., Xia T.-L., Tang H.-C., Liu X., Han R., Zou X., Zhao Y.-T., Chen M.-Y., Li G. (2022). Establishment of a patient-derived organoid model and living biobank for nasopharyngeal carcinoma. Ann. Transl. Med..

[74] Hui K.P.Y., Ching R.H.H., Chan S.K.H., Nicholls J.M., Sachs N., Clevers H., Peiris J.S.M., Chan M.C.W. (2018). Tropism, replication competence, and innate immune responses of influenza virus: An analysis of human airway organoids and ex-vivo bronchus cultures. Lancet Respir. Med..

[75] Li L., Jiao L., Feng D., Yuan Y., Yang X., Li J., Jiang D., Chen H., Meng Q., Chen R. (2024). Human apical-out nasal organoids reveal an essential role of matrix metalloproteinases in airway epithelial differentiation. Nat. Commun..

[76] Kumar P.A., Hu Y., Yamamoto Y., Hoe N.B., Wei T.S., Mu D., Sun Y., Joo L.S., Dagher R., Zielonka E.M. (2011). Distal Airway Stem Cells Yield Alveoli In Vitro and during Lung Regeneration following H1N1 Influenza Infection. Cell.

[77] Konishi S., Gotoh S., Tateishi K., Yamamoto Y., Korogi Y., Nagasaki T., Matsumoto H., Muro S., Hirai T., Ito I. (2016). Directed Induction of Functional Multi-ciliated Cells in Proximal Airway Epithelial Spheroids from Human Pluripotent Stem Cells. Stem Cell Rep..

[78] Miller A.J., Dye B.R., Ferrer-Torres D., Hill D.R., Overeem A.W., Shea L.D., Spence J.R. (2019). Generation of lung organoids from human pluripotent stem cells in vitro. Nat. Protoc..

[79] Miller A.J., Yu Q., Czerwinski M., Tsai Y.-H., Conway R.F., Wu A., Holloway E.M., Walker T., Glass I.A., Treutlein B. (2020). In Vitro and In Vivo Development of the Human Airway at Single-Cell Resolution. Dev. Cell.

[80] Nikolić M.Z., Caritg O., Jeng Q., Johnson J.-A., Sun D., Howell K.J., Brady J.L., Laresgoiti U., Allen G., Butler R. (2017). Human embryonic lung epithelial tips are multipotent progenitors that can be expanded in vitro as long-term self-renewing organoids. eLife.

[81] Hein R.F., Wu J.H., Holloway E.M., Frum T., Conchola A.S., Tsai Y.-H., Wu A., Fine A.S., Miller A.J., Szenker-Ravi E. (2022). R-SPONDIN2 mesenchymal cells form the bud tip progenitor niche during human lung development. Dev. Cell.

[82] Tan Q., Choi K.M., Sicard D., Tschumperlin D.J. (2017). Human airway organoid engineering as a step toward lung regeneration and disease modeling. Biomaterials.

[83] Sachs N., Papaspyropoulos A., Zomer-van Ommen D.D., Heo I., Böttinger L., Klay D., Weeber F., Huelsz-Prince G., Iakobachvili N., Amatngalim G.D. (2019). Long-term expanding human airway organoids for disease modeling. EMBO J..

[84] Mou H., Vinarsky V., Tata P.R., Brazauskas K., Choi S., Crooke A.K., Zhang B., Solomon G.M., Turner B., Bihler H. (2016). Dual SMAD Signaling Inhibition Enables Long-Term Expansion of Diverse Epithelial Basal Cells. Cell Stem Cell.

[85] Moiseenko A., Vazquez-Armendariz A.I., Kheirollahi V., Chu X., Tata A., Rivetti S., Günther S., Lebrigand K., Herold S., Braun T. (2020). Identification of a Repair-Supportive Mesenchymal Cell Population during Airway Epithelial Regeneration. Cell Rep..

[86] Vazquez-Armendariz A.I., Seeger W., Herold S., El Agha E. (2021). Protocol for the generation of murine bronchiolospheres. STAR Protoc..

[87] Lee J.-H., Bhang D.H., Beede A., Huang T.L., Stripp B.R., Bloch K.D., Wagers A.J., Tseng Y.-H., Ryeom S., Kim C.F. (2014). Lung Stem Cell Differentiation in Mice Directed by Endothelial Cells via a BMP4-NFATc1-Thrombospondin-1 Axis. Cell.

[88] Raasch K., Maurat E., Henrot P., Zysman M., Prevel R., Thumerel M., Nassoy P., Berger P., Andrique L., Recher G. (2024). A novel in vitro tubular model to recapitulate features of distal airways: The bronchioid. Rev. Des Mal. Respir..

[89] Liu M.Y., Chen B., Borji M., Rivas C.G.d.A., Dost A.F.M., Moye A.L., Abdulla N.M., Paschini M., Rollins S.D., Wang R. (2024). Human Airway and Alveolar Organoids from BAL Fluid. Am. J. Respir. Crit. Care Med..

[90] Chiu M.C., Zhang S., Li C., Liu X., Yu Y., Huang J., Wan Z., Zhu X., Zhou J. (2023). Apical-Out Human Airway Organoids Modeling SARS-CoV-2 Infection. Viruses.

[91] Zacharias W.J., Frank D.B., Zepp J.A., Morley M.P., Alkhaleel F.A., Kong J., Zhou S., Cantu E., Morrisey E.E. (2018). Regeneration of the lung alveolus by an evolutionarily conserved epithelial progenitor. Nature.

[92] Leeman K.T., Pessina P., Lee J.-H., Kim C.F. (2019). Mesenchymal Stem Cells Increase Alveolar Differentiation in Lung Progenitor Organoid Cultures. Sci. Rep..

[93] Hoffmann K., Obermayer B., Hönzke K., Fatykhova D., Demir Z., Löwa A., Alves L.G.T., Wyler E., Lopez-Rodriguez E., Mieth M. (2022). Human alveolar progenitors generate dual lineage bronchioalveolar organoids. Commun. Biol..

[94] Wu D., Pan W. (2010). GSK3: A multifaceted kinase in Wnt signaling. Trends Biochem. Sci..

[95] Shiraishi K., Nakajima T., Shichino S., Deshimaru S., Matsushima K., Ueha S. (2019). In vitro expansion of endogenous human alveolar epithelial type II cells in fibroblast-free spheroid culture. Biochem. Biophys. Res. Commun..

[96] Liu K., Tang M., Liu Q., Han X., Jin H., Zhu H., Li Y., He L., Ji H., Zhou B. (2020). Bi-directional differentiation of single bronchioalveolar stem cells during lung repair. Cell Discov..

[97] Vazquez-Armendariz A.I., Heiner M., El Agha E., Salwig I., Hoek A., Hessler M.C., Shalashova I., Shrestha A., Carraro G., Mengel J.P. (2020). Multilineage murine stem cells generate complex organoids to model distal lung development and disease. EMBO J..

[98] Salahudeen A.A., Choi S.S., Rustagi A., Zhu J., van Unen V., de la O S.M., Flynn R.A., Margalef-Català M., Santos A.J.M., Ju J. (2020). Progenitor identification and SARS-CoV-2 infection in human distal lung organoids. Nature.

[99] Dye B.R., Hill D.R., Ferguson M.A., Tsai Y.-H., Nagy M.S., Dyal R., Wells J.M., Mayhew C.N., Nattiv R., Klein O.D. (2015). In vitro generation of human pluripotent stem cell derived lung organoids. eLife.

[100] Tamò L., Hibaoui Y., Kallol S., Alves M.P., Albrecht C., Hostettler K.E., Feki A., Rougier J.-S., Abriel H., Knudsen L. (2018). Generation of an alveolar epithelial type II cell line from induced pluripotent stem cells. Am. J. Physiol. Cell. Mol. Physiol..

[101] Ozan V.B., Wang H., Akshay A., Anand D., Hibaoui Y., Feki A., Gote-Schniering J., Gheinani A.H., Heller M., Uldry A.-C. (2024). Influence of Microenvironmental Orchestration on Multicellular Lung Alveolar Organoid Development from Human Induced Pluripotent Stem Cells. Stem Cell Rev. Rep..

[102] Lee J., Baek H., Jang J., Park J., Cha S.-R., Hong S.-H., Kim J., Lee J.-H., Hong I.-S., Wang S.-J. (2023). Establishment of a human induced pluripotent stem cell derived alveolar organoid for toxicity assessment. Toxicol. In Vitro.

[103] Seo H.-R., Han H.-J., Lee Y., Noh Y.-W., Cho S.-J., Kim J.-H. (2022). Human Pluripotent Stem Cell-Derived Alveolar Organoid with Macrophages. Int. J. Mol. Sci..

[104] Yamamoto Y., Gotoh S., Korogi Y., Seki M., Konishi S., Ikeo S., Sone N., Nagasaki T., Matsumoto H., Muro S. (2017). Long-term expansion of alveolar stem cells derived from human iPS cells in organoids. Nat. Methods.

[105] Tamai K., Sakai K., Yamaki H., Moriguchi K., Igura K., Maehana S., Suezawa T., Takehara K., Hagiwara M., Hirai T. (2022). iPSC-derived mesenchymal cells that support alveolar organoid development. Cell Rep. Methods.

[106] Purev E., Bahmed K., Kosmider B. (2024). Alveolar Organoids in Lung Disease Modeling. Biomolecules.

[107] Chen J., Na F. (2022). Organoid technology and applications in lung diseases: Models, mechanism research and therapy opportunities. Front. Bioeng. Biotechnol..

[108] Lee J., Kim J.-H., Hong S.-H., Yang S.-R. (2021). Organoid Model in Idiopathic Pulmonary Fibrosis. Int. J. Stem Cells.

[109] Kim J.-H., An G.H., Kim J.-Y., Rasaei R., Kim W.J., Jin X., Woo D.-H., Han C., Yang S.-R., Kim J.-H. (2021). Human pluripotent stem cell-derived alveolar organoids for modeling pulmonary fibrosis and drug testing. Cell Death Discov..

[110] Chen Y.-W., Huang S.X., de Carvalho A.L.R.T., Ho S.-H., Islam M.N., Volpi S., Notarangelo L.D., Ciancanelli M., Casanova J.-L., Bhattacharya J. (2017). A three-dimensional model of human lung development and disease from pluripotent stem cells. Nat. Cell Biol..

[111] Surolia R., Li F.J., Wang Z., Li H., Liu G., Zhou Y., Luckhardt T., Bae S., Liu R.-M., Rangarajan S. (2017). 3D pulmospheres serve as a personalized and predictive multicellular model for assessment of antifibrotic drugs. J. Clin. Investig..

[112] Strikoudis A., Cieślak A., Loffredo L., Chen Y.-W., Patel N., Saqi A., Lederer D.J., Snoeck H.-W. (2019). Modeling of Fibrotic Lung Disease Using 3D Organoids Derived from Human Pluripotent Stem Cells. Cell Rep..

[113] Xu C., Zhao J., Li Q., Hou L., Wang Y., Li S., Jiang F., Zhu Z., Tian L. (2020). Exosomes derived from three-dimensional cultured human umbilical cord mesenchymal stem cells ameliorate pulmonary fibrosis in a mouse silicosis model. Stem Cell Res. Ther..

[114] Ptasinski V., Monkley S.J., Öst K., Tammia M., Alsafadi H.N., Overed-Sayer C., Hazon P., E Wagner D., A Murray L. (2023). Modeling fibrotic alveolar transitional cells with pluripotent stem cell-derived alveolar organoids. Life Sci. Alliance.

[115] Wang S., Li X., Ma Q., Wang Q., Wu J., Yu H., Li K., Li Y., Wang J., Zhang Q. (2022). Glutamine Metabolism Is Required for Alveolar Regeneration during Lung Injury. Biomolecules.

[116] Suezawa T., Kanagaki S., Moriguchi K., Masui A., Nakao K., Toyomoto M., Tamai K., Mikawa R., Hirai T., Murakami K. (2021). Disease modeling of pulmonary fibrosis using human pluripotent stem cell-derived alveolar organoids. Stem Cell Rep..

[117] Sundarakrishnan A., Chen Y., Black L.D., Aldridge B.B., Kaplan D.L. (2018). Engineered cell and tissue models of pulmonary fibrosis. Adv. Drug Deliv. Rev..

[118] Wilkinson D.C., Mellody M., Meneses L.K., Hope A.C., Dunn B., Gomperts B.N. (2018). Development of a Three-Dimensional Bioengineering Technology to Generate Lung Tissue for Personalized Disease Modeling. Curr. Protoc. Stem Cell Biol..

[119] Jaeger B., Schupp J.C., Plappert L., Terwolbeck O., Artysh N., Kayser G., Engelhard P., Adams T.S., Zweigerdt R., Kempf H. (2022). Airway basal cells show a dedifferentiated KRT17highPhenotype and promote fibrosis in idiopathic pulmonary fibrosis. Nat. Commun..

[120] Huang G., Liang J., Huang K., Liu X., Taghavifar F., Yao C., Parimon T., Liu N., Dai K., Aziz A. (2023). Basal Cell–derived WNT7A Promotes Fibrogenesis at the Fibrotic Niche in Idiopathic Pulmonary Fibrosis. Am. J. Respir. Cell Mol. Biol..

[121] Enomoto Y., Katsura H., Fujimura T., Ogata A., Baba S., Yamaoka A., Kihara M., Abe T., Nishimura O., Kadota M. (2023). Autocrine TGF-β-positive feedback in profibrotic AT2-lineage cells plays a crucial role in non-inflammatory lung fibrogenesis. Nat. Commun..

[122] Sui J., Boatz J.C., Shi J., Hu Q., Li X., Zhang Y., Königshoff M., Kliment C.R. (2023). Loss of ANT1 Increases Fibrosis and Epithelial Cell Senescence in Idiopathic Pulmonary Fibrosis. Am. J. Respir. Cell Mol. Biol..

[123] Cohen M.L., Brumwell A.N., Ho T.C., Garakani K., Montas G., Leong D., Ding V.W., Golden J.A., Trinh B.N., Jablons D.M. (2024). A fibroblast-dependent TGF-β1/sFRP2 noncanonical Wnt signaling axis promotes epithelial metaplasia in idiopathic pulmonary fibrosis. J. Clin. Investig..

[124] Wu X., Verschut V., Woest M.E., Ng-Blichfeldt J.-P., Matias A., Villetti G., Accetta A., Facchinetti F., Gosens R., Kistemaker L.E.M. (2021). Rho-Kinase 1/2 Inhibition Prevents Transforming Growth Factor-β-Induced Effects on Pulmonary Remodeling and Repair. Front. Pharmacol..

[125] Wang H., Han Z., Yang Y., Liu L., Huang Y., Chen J., Wang Y., Liu Z., Xin L., Zhao Y. (2025). Modeling of lung organoid-based fibrosis for testing the sensitivity of anti-fibrotic drugs. Stem Cell Res. Ther..

[126] Marwick J.A., Elliott R.J.R., Longden J., Makda A., Hirani N., Dhaliwal K., Dawson J.C., Carragher N.O. (2022). Application of a High-Content Screening Assay Utilizing Primary Human Lung Fibroblasts to Identify Antifibrotic Drugs for Rapid Repurposing in COVID-19 Patients. SLAS Discov. Adv. Sci. Drug Discov..

[127] Calucho M., Gartner S., Barranco P., Fernández-Álvarez P., Pérez R.G., Tizzano E.F. (2021). Validation of nasospheroids to assay CFTR functionality and modulator responses in cystic fibrosis. Sci. Rep..

[128] McCauley K.B., Hawkins F., Serra M., Thomas D.C., Jacob A., Kotton D.N. (2017). Efficient Derivation of Functional Human Airway Epithelium from Pluripotent Stem Cells via Temporal Regulation of Wnt Signaling. Cell Stem Cell.

[129] Dekkers J.F., Berkers G., Kruisselbrink E., Vonk A., de Jonge H.R., Janssens H.M., Bronsveld I., van de Graaf E.A., Nieuwenhuis E.E.S., Houwen R.H.J. (2016). Characterizing responses to CFTR-modulating drugs using rectal organoids derived from subjects with cystic fibrosis. Sci. Transl. Med..

[130] Ramalho A.S., Fürstová E., Vonk A.M., Ferrante M., Verfaillie C., Dupont L., Boon M., Proesmans M., Beekman J.M., Sarouk I. (2020). Correction of CFTR function in intestinal organoids to guide treatment of cystic fibrosis. Eur. Respir. J..

[131] Guimbellot J.S., Leach J.M., Chaudhry I.G., Quinney N.L., Boyles S.E., Chua M., Aban I., Jaspers I., Gentzsch M. (2017). Nasospheroids permit measurements of CFTR-dependent fluid transport. J. Clin. Investig..

[132] Liu Z., Anderson J.D., Deng L., Mackay S., Bailey J., Kersh L., Rowe S.M., Guimbellot J.S. (2020). Human Nasal Epithelial Organoids for Therapeutic Development in Cystic Fibrosis. Genes.

[133] Sette G., Lo Cicero S., Blaconà G., Pierandrei S., Bruno S.M., Salvati V., Castelli G., Falchi M., Fabrizzi B., Cimino G. (2021). Theratyping cystic fibrosis in vitro in ALI culture and organoid models generated from patient-derived nasal epithelial conditionally reprogrammed stem cells. Eur. Respir. J..

[134] Amatngalim G.D., Rodenburg L.W., Aalbers B.L., Raeven H.H., Aarts E.M., Sarhane D., Spelier S., Lefferts J.W., AL Silva I., Nijenhuis W. (2022). Measuring cystic fibrosis drug responses in organoids derived from 2D differentiated nasal epithelia. Life Sci. Alliance.

[135] Keating D., Marigowda G., Burr L., Daines C., Mall M.A., McKone E.F., Ramsey B.W., Rowe S.M., Sass L.A., Tullis E. (2018). VX-445-Tezacaftor-Ivacaftor in Patients with Cystic Fibrosis and One or Two Phe508del Alleles. N. Engl. J. Med..

[136] Anderson J.D., Liu Z., Odom L.V., Kersh L., Guimbellot J.S. (2021). CFTR function and clinical response to modulators parallel nasal epithelial organoid swelling. Am. J. Physiol. Cell. Mol. Physiol..

[137] Geurts M.H., de Poel E., Amatngalim G.D., Oka R., Meijers F.M., Kruisselbrink E., van Mourik P., Berkers G., Groot K.M.d.W.-D., Michel S. (2020). CRISPR-Based Adenine Editors Correct Nonsense Mutations in a Cystic Fibrosis Organoid Biobank. Cell Stem Cell.

[138] Wu X., Bos I.S.T., Conlon T.M., Ansari M., Verschut V., van der Koog L., Verkleij L.A., D’aMbrosi A., Matveyenko A., Schiller H.B. (2022). A transcriptomics-guided drug target discovery strategy identifies receptor ligands for lung regeneration. Sci. Adv..

[139] Hu Y., Ng-Blichfeldt J.-P., Ota C., Ciminieri C., Ren W., Hiemstra P.S., Stolk J., Gosens R., Königshoff M. (2020). Wnt/β-catenin signaling is critical for regenerative potential of distal lung epithelial progenitor cells in homeostasis and emphysema. STEM CELLS.

[140] Chan L.L.Y., Anderson D.E., Cheng H.S., Ivan F.X., Chen S., Kang A.E.Z., Foo R., Gamage A.M., Tiew P.Y., Koh M.S. (2022). The establishment of COPD organoids to study host-pathogen interaction reveals enhanced viral fitness of SARS-CoV-2 in bronchi. Nat. Commun..

[141] Li Y., He Y., Zheng Q., Zhang J., Pan X., Zhang X., Yuan H., Wang G., Liu X., Zhou X. (2024). Mitochondrial pyruvate carriers control airway basal progenitor cell function through glycolytic-epigenetic reprogramming. Cell Stem Cell.

[142] Bernardo M.E., Fibbe W.E. (2013). Mesenchymal Stromal Cells: Sensors and Switchers of Inflammation. Cell Stem Cell.

[143] Weiss A.R.R., Dahlke M.H. (2019). Immunomodulation by Mesenchymal Stem Cells (MSCs): Mechanisms of Action of Living, Apoptotic, and Dead MSCs. Front. Immunol..

[144] Reinders M.E., de Fijter J.W., Roelofs H., Bajema I.M., de Vries D.K., Schaapherder A.F., Claas F.H., van Miert P.P., Roelen D.L., van Kooten C. (2013). Autologous Bone Marrow-Derived Mesenchymal Stromal Cells for the Treatment of Allograft Rejection After Renal Transplantation: Results of a Phase I Study. STEM CELLS Transl. Med..

[145] Rodrigo S.F., van Ramshorst J., Hoogslag G.E., Boden H., Velders M.A., Cannegieter S.C., Roelofs H., Al Younis I., Dibbets-Schneider P., Fibbe W.E. (2013). Intramyocardial Injection of Autologous Bone Marrow-Derived Ex Vivo Expanded Mesenchymal Stem Cells in Acute Myocardial Infarction Patients is Feasible and Safe up to 5 Years of Follow-up. J. Cardiovasc. Transl. Res..

[146] Kruk D.M.L.W., Wisman M., Noordhoek J.A., Nizamoglu M., Jonker M.R., de Bruin H.G., Gomez K.A., Hacken N.H.T.T., Pouwels S.D., Heijink I.H. (2021). Paracrine Regulation of Alveolar Epithelial Damage and Repair Responses by Human Lung-Resident Mesenchymal Stromal Cells. Cells.

[147] Skronska-Wasek W., Mutze K., Baarsma H.A., Bracke K.R., Alsafadi H.N., Lehmann M., Costa R., Stornaiuolo M., Novellino E., Brusselle G.G. (2017). Reduced Frizzled Receptor 4 Expression Prevents WNT/β-Catenin–driven Alveolar Lung Repair in Chronic Obstructive Pulmonary Disease. Am. J. Respir. Crit. Care Med..

[148] Basil M.C., Cardenas-Diaz F.L., Kathiriya J.J., Morley M.P., Carl J., Brumwell A.N., Katzen J., Slovik K.J., Babu A., Zhou S. (2022). Human distal airways contain a multipotent secretory cell that can regenerate alveoli. Nature.

[149] Costa R., Wagner D.E., Doryab A., De Santis M.M., Schorpp K., Rothenaigner I., Lehmann M., Baarsma H.A., Liu X., Schmid O. (2021). A drug screen with approved compounds identifies amlexanox as a novel Wnt/β-catenin activator inducing lung epithelial organoid formation. Br. J. Pharmacol..

[150] Pardal R., Clarke M.F., Morrison S.J. (2003). Applying the principles of stem-cell biology to cancer. Nat. Rev. Cancer.

[151] Zhang D.-G., Jiang A.-G., Lu H.-Y., Zhang L.-X., Gao X.-Y. (2014). Isolation, cultivation and identification of human lung adenocarcinoma stem cells. Oncol. Lett..

[152] Di Liello R., Ciaramella V., Barra G., Venditti M., Della Corte C.M., Papaccio F., Sparano F., Viscardi G., Iacovino M.L., Minucci S. (2019). Ex vivo lung cancer spheroids resemble treatment response of a patient with NSCLC to chemotherapy and immunotherapy: Case report and translational study. ESMO Open.

[153] Zhang Z., Wang H., Ding Q., Xing Y., Xu Z., Lu C., Luo D., Xu L., Xia W., Zhou C. (2018). Establishment of patient-derived tumor spheroids for non-small cell lung cancer. PLOS ONE.

[154] Weeber F., van de Wetering M., Hoogstraat M., Dijkstra K.K., Krijgsman O., Kuilman T., Gadellaa-van Hooijdonk C.G.M., van der Velden D.L., Peeper D.S., Cuppen E.P.J.G. (2015). Preserved genetic diversity in organoids cultured from biopsies of human colorectal cancer metastases. Proc. Natl. Acad. Sci. USA.

[155] Park J.W., Lee J.K., Sheu K.M., Wang L., Balanis N.G., Nguyen K., Smith B.A., Cheng C., Tsai B.L., Cheng D. (2018). Reprogramming normal human epithelial tissues to a common, lethal neuroendocrine cancer lineage. Science.

[156] Kim M., Mun H., Sung C.O., Cho E.J., Jeon H.-J., Chun S.-M., Jung D.J., Shin T.H., Jeong G.S., Kim D.K. (2019). Patient-derived lung cancer organoids as in vitro cancer models for therapeutic screening. Nat. Commun..

[157] Richer A.L., Cala J.M., O’Brien K., Carson V.M., Inge L.J., Whitsett T.G. (2017). WEE1 Kinase Inhibitor AZD1775 Has Preclinical Efficacy in LKB1-Deficient Non–Small Cell Lung Cancer. Cancer Res..

[158] Zhang H., Brainson C.F., Koyama S., Redig A.J., Chen T., Li S., Gupta M., Garcia-De-Alba C., Paschini M., Herter-Sprie G.S. (2017). Lkb1 inactivation drives lung cancer lineage switching governed by Polycomb Repressive Complex 2. Nat. Commun..

[159] Lazarus K.A., Hadi F., Zambon E., Bach K., Santolla M.-F., Watson J.K., Correia L.L., Das M., Ugur R., Pensa S. (2018). BCL11A interacts with SOX2 to control the expression of epigenetic regulators in lung squamous carcinoma. Nat. Commun..

[160] Caponigro G., Sellers W.R. (2011). Advances in the preclinical testing of cancer therapeutic hypotheses. Nat. Rev. Drug Discov..

[161] Barrera-Rodríguez R., Fuentes J.M. (2015). Multidrug resistance characterization in multicellular tumour spheroids from two human lung cancer cell lines. Cancer Cell Int..

[162] Hai J., Zhang H., Zhou J., Wu Z., Chen T., Papadopoulos E., Dowling C.M., Pyon V., Pan Y., Bin Liu J. (2020). Generation of Genetically Engineered Mouse Lung Organoid Models for Squamous Cell Lung Cancers Allows for the Study of Combinatorial Immunotherapy. Clin. Cancer Res..

[163] Wang Y., Jiang T., Qin Z., Jiang J., Wang Q., Yang S., Rivard C., Gao G., Ng T., Tu M. (2019). HER2 exon 20 insertions in non-small-cell lung cancer are sensitive to the irreversible pan-HER receptor tyrosine kinase inhibitor pyrotinib. Ann. Oncol..

[164] Takahashi N., Hoshi H., Higa A., Hiyama G., Tamura H., Ogawa M., Takagi K., Goda K., Okabe N., Muto S. (2019). An In Vitro System for Evaluating Molecular Targeted Drugs Using Lung Patient-Derived Tumor Organoids. Cells.

[165] Dijkstra K.K., Cattaneo C.M., Weeber F., Chalabi M., Van De Haar J., Fanchi L.F., Slagter M., Van Der Velden D.L., Kaing S., Kelderman S. (2018). Generation of Tumor-Reactive T Cells by Co-culture of Peripheral Blood Lymphocytes and Tumor Organoids. Cell.

[166] Sahin U. (2018). Studying Tumor-ReacTive T Cells: A Personalized Organoid Model. Cell Stem Cell.

[167] He X., Xu C. (2020). Immune checkpoint signaling and cancer immunotherapy. Cell Res..

[168] Yui S., Nakamura T., Sato T., Nemoto Y., Mizutani T., Zheng X., Ichinose S., Nagaishi T., Okamoto R., Tsuchiya K. (2012). Functional engraftment of colon epithelium expanded in vitro from a single adult Lgr5+ stem cell. Nat. Med..

[169] Yin X., Mead B.E., Safaee H., Langer R., Karp J.M., Levy O. (2016). Engineering Stem Cell Organoids. Cell Stem Cell.

[170] Miura Y., Li M.-Y., Birey F., Ikeda K., Revah O., Thete M.V., Park J.-Y., Puno A., Lee S.H., Porteus M.H. (2020). Generation of human striatal organoids and cortico-striatal assembloids from human pluripotent stem cells. Nat. Biotechnol..

[171] Xiang Y., Tanaka Y., Patterson B., Kang Y.-J., Govindaiah G., Roselaar N., Cakir B., Kim K.-Y., Lombroso A.P., Hwang S.-M. (2017). Fusion of Regionally Specified hPSC-Derived Organoids Models Human Brain Development and Interneuron Migration. Cell Stem Cell.

[172] Bin Ramli M.N., Lim Y.S., Koe C.T., Demircioglu D., Tng W., Gonzales K.A.U., Tan C.P., Szczerbinska I., Liang H., Soe E.L. (2020). Human Pluripotent Stem Cell-Derived Organoids as Models of Liver Disease. Gastroenterology.

[173] Koike H., Iwasawa K., Ouchi R., Maezawa M., Giesbrecht K., Saiki N., Ferguson A., Kimura M., Thompson W.L., Wells J.M. (2019). Modelling human hepato-biliary-pancreatic organogenesis from the foregut–midgut boundary. Nature.

[174] Shah V.S., Chivukula R.R., Lin B., Waghray A., Rajagopal J. (2022). Cystic Fibrosis and the Cells of the Airway Epithelium: What Are Ionocytes and What Do They Do?. Annu. Rev. Pathol. Mech. Dis..

[175] Lamers M.M., van der Vaart J., Knoops K., Riesebosch S., Breugem T.I., Mykytyn A.Z., Beumer J., Schipper D., Bezstarosti K., Koopman C.D. (2021). An organoid-derived bronchioalveolar model for SARS-CoV-2 infection of human alveolar type II-like cells. EMBO J..

[176] Wang J., Li X., Chen H. (2020). Organoid models in lung regeneration and cancer. Cancer Lett..

[177] Zhang C., Zhao Z., Rahim N.A.A., van Noort D., Yu H. (2009). Towards a human-on-chip: Culturing multiple cell types on a chip with compartmentalized microenvironments. Lab Chip.

[178] Daly A.C., Davidson M.D., Burdick J.A. (2021). 3D bioprinting of high cell-density heterogeneous tissue models through spheroid fusion within self-healing hydrogels. Nat. Commun..

[179] Quadrato G., Nguyen T., Macosko E.Z., Sherwood J.L., Min Yang S., Berger D.R., Maria N., Scholvin J., Goldman M., Kinney J.P. (2017). Cell diversity and network dynamics in photosensitive human brain organoids. Nature.

[180] Ng W.H., Johnston E.K., Tan J.J., Bliley J.M., Feinberg A.W., Stolz D.B., Sun M., Wijesekara P., Hawkins F., Kotton D.N. (2022). Recapitulating human cardio-pulmonary co-development using simultaneous multilineage differentiation of pluripotent stem cells. eLife.

[181] Qadir A.S., Das S., Nedunchezian S., Masuhara K., Desai T.J., Rehman J., Murthy P.K., Tsukasaki Y., Shao L., Malik A.B. (2025). Physiological Modeling of the Vascularized Human Lung Organoid. Am. J. Respir. Cell Mol. Biol..

[182] Jiang S., Chen D., Tian L., Pan Z., Long H., Chu L., Kong W., Yao Q., Ma X., Zhao Y. (2025). Role of BMPR2 Mutation in Lung Organoid Differentiation. Biomedicines.

[183] Huskin G., Chen J., Davis T., Jun H.-W. (2023). Tissue-Engineered 3D In Vitro Disease Models for High-Throughput Drug Screening. Tissue Eng. Regen. Med..

